# Psychological Distance to Science as a Predictor of Science Skepticism Across Domains

**DOI:** 10.1177/01461672221118184

**Published:** 2022-09-03

**Authors:** Bojana Većkalov, Natalia Zarzeczna, Jonathon McPhetres, Frenk van Harreveld, Bastiaan T. Rutjens

**Affiliations:** 1 University of Amsterdam, The Netherlands; 2 Durham University, UK

**Keywords:** science skepticism, public perceptions of science, psychological distance, psychological distance to science, scale construction

## Abstract

This article presents and tests psychological distance to science (PSYDISC) as a domain-general predictor of science skepticism. Drawing on the concept of psychological distance, PSYDISC reflects the extent to which individuals perceive science as a tangible undertaking conducted by people similar to oneself (*social*), with effects in the here (*spatial*) and now (*temporal*), and as useful and applicable in the real world (*hypothetical distance*). In six studies (two preregistered; total *N* = 1,630) and two countries, we developed and established the factor structure and validity of a scale measuring PSYDISC. Crucially, higher PSYDISC predicted skepticism beyond established predictors, across science domains. A final study showed that PSYDISC shapes real-world behavior (COVID-19 vaccination uptake). This work thus provides a novel tool to predict science skepticism, as well as a construct that can help to further develop a unifying framework to understand science skepticism across domains.

Trust in science among the public is generally quite high, as indicated by recent survey results showing that 90% of people across 17 countries (somewhat) agree with the statement “I trust science.” However, at the same time, 40% of the same people (somewhat) agree with the statement “I only believe science that aligns with my personal beliefs” (3M, 2021). This contrast provides a glimpse into the mismatch between trust in science in general and skepticism about specific science domains. Indeed, public opinion is often at odds with the scientific consensus—for example, a survey from 2014 found that while 93% of Earth scientists agreed that human activity is a major contributor to climate change, only 50% of the public agreed with this. Likewise, 87% of U.S. biomedical scientists stated that childhood vaccines should be required, in contrast to 68% of the public (Funk & Rainie, 2015). A similar gulf between scientific evidence and public acceptance has been observed during the COVID-19 pandemic (Rothmund et al., 2022). Such science skepticism—which we define as the systematic and unwarranted rejection of empirical evidence or well-established scientific findings (see, for example, Rutjens et al., 2022)—can have damaging consequences for individual and environmental health, for example, when it leads to a lack of public support for action in the case of climate change (Gifford, 2011; Steffen et al., 2018; van der Linden et al., 2015) and insufficient vaccination rates (Betsch et al., 2010; Roozenbeek et al., 2020; van Panhuis et al., 2013).

Even though research into science skepticism has been rapidly developing in recent years, state-of-the art understanding of psychological factors contributing to science skepticism remains somewhat fragmented and limited. This is partly because attitudes to various science topics are often studied in isolation (but see Drummond & Fischhoff, 2017b; Lewandowsky et al., 2013; Rutjens et al., 2022), making comparisons and translating insights from one domain to another difficult. In addition, most research on science skepticism can be divided into two streams. The first is focused on identifying antecedents such as ideologies, values, worldviews, identities, and other underlying *motivations* for rejecting science (Hornsey & Fielding, 2017; Rutjens et al., 2018). The second stream builds on the broadly defined “deficit model” of science communication (e.g., Sturgis & Allum, 2004). This model assumes that the general public lacks *information*, and subsequently knowledge, about science, and that this is the main driver of science skepticism. Although the original deficit account has been largely disputed (e.g., Nisbet & Scheufele, 2009; Simis et al., 2016), its influence is noticeable in more recent work emphasizing the roles of cognitive sophistication (Pennycook et al., 2022) and accuracy motives (Pennycook et al., 2020) in science skepticism. Although both streams have contributed to understanding science skepticism and should be seen as complementary (van der Linden & Lewandowsky, 2022; Zarzeczna et al., 2021), they mostly focus on antecedents of science skepticism which are (a) difficult to change and (b) domain-specific.

First, ideological predictors (i.e., motivational underpinnings of science skepticism) constitute largely stable beliefs that cannot be easily influenced. Furthermore, while increasing knowledge and providing accurate information about science is theoretically possible, at scale, it can be unfeasible to sufficiently increase science understanding. Moreover, many forms of skepticism seem unrelated or only very weakly related to science literacy (see Rutjens et al., 2018, 2022), and emphasizing the value of agreed upon knowledge (i.e., scientific consensus) in a particular domain is not always useful. More specifically, this has been shown to be effective in the case of genetically modified (GM) foods, but not climate change (van Stekelenburg et al., 2021). Finally, in some instances, increased knowledge and reasoning ability can even facilitate the “bending” of science information to fit the individual’s ideology or worldview (e.g., Drummond & Fischhoff, 2017b; Kahan et al., 2012).

Second, public opinion on specific science topics—such as climate change, vaccination, or evolution—is associated with different individual difference factors, pointing to the heterogenous nature of science skepticism. For example, climate change skepticism is highly contingent on political ideology (Hornsey et al., 2016; Rutjens et al., 2022). In contrast, political ideology is not clearly associated with vaccine skepticism, which involves religious and spiritual beliefs, science knowledge, and conspiratorial thinking (Hornsey et al., 2018; Rutjens et al., 2021, 2022; Rutjens & van der Lee, 2020). Evolution skepticism has religious orthodoxy as its strongest antecedent (Rutjens et al., 2022; Rutjens & van der Lee, 2020), while ideologies and worldviews do not play a consistent role in GM food skepticism, but science knowledge does (McPhetres et al., 2019; Rutjens et al., 2022).

In this article, we introduce and test a construct that goes beyond the domain-specific, immutable, and mostly descriptive determinants of science skepticism. To do so, we apply the construct of psychological distance—“a subjective experience that something is close or far away from the self, here, and now” (Trope & Liberman, 2010)—to perceptions of science. More specifically, we propose that perceived psychological distance to science (PSYDISC) in temporal, spatial, social, and hypothetical terms contributes to science skepticism across various science domains (i.e., in this article, these are climate change, vaccination, evolution, genetic modification of food, and genetic editing in humans). We hypothesize that PSYDISC explains unique variance over and above domain-specific demographic, ideological, and knowledge antecedents. As such, PSYDISC offers a more comprehensive approach, as it provides an understanding of science skepticism *across* science domains. In addition, this approach could also be leveraged to inform interventions aimed at countering science rejection, as psychological distance (or proximity) toward a specific domain or finding can be easily incorporated in science communication efforts (Zarzeczna et al., 2022). Next, we describe the concept of psychological distance to science in more detail.

## Psychological Distance to Science (PSYDISC)

Psychological distance to science refers to perceptions of science in terms of its tangibility and relevance for the individual. In other words, PSYDISC pertains to how one evaluates science from the perspective of the self. Lower psychological distance (i.e., psychological proximity) entails that science—and scientific research—is perceived as a tangible undertaking with effects that bear relevance to the individual. This perceived closeness to science is reflected in four psychological distance dimensions, which stem from Construal Level Theory (CLT; Liberman & Trope, 2014; Trope & Liberman, 2010), are interrelated (Fiedler et al., 2012) and share a common meaning of distancing from direct experience (Maglio et al., 2013). More specifically, psychological proximity to science entails perceiving it as relevant for the local community (i.e., spatial) and for the present time (i.e., temporal). In addition, proximity to science involves perceiving it as tangible, in terms of it having practical implications and tangible effects on the world (i.e., hypothetical), as well as it being conducted by individuals that are approachable and similar to oneself (i.e., social proximity). It is likely, however, that, to many people, science does not have that meaning (e.g., Humm & Schrögel, 2020; Wellcome Global Monitor, 2018). This could be due to, for example, a lack of exposure to science or interaction with scientists and scientific content. To these individuals, science will feel more psychologically distant; that is, as an unclear process with no direct relevance to one’s life. PSYDISC proposes that such psychological distance to science is related to—and facilitates—science skepticism across domains.

Although this work is the first to investigate psychological distance to science as a precursor of science skepticism, previous research provides indirect support for our assumptions. First, work on public engagement with climate change shows that perceiving climate change as psychologically distant (i.e., as a problem that affects distant places, distant and dissimilar people, and may or may not occur sometime in the future, in the form of some uncertain set of events) relates to less perceived relevance (Loy & Spence, 2020; Spence & Pidgeon, 2010) and consequently more skepticism and less concern about the issue (Spence et al., 2012; Većkalov et al., 2021; Wang et al., 2019). Although this research focused on distance to the environmental and societal *consequences* of climate change rather than distance to climate *science*, it does demonstrate the general potential of psychological distance to predict attitudes toward science topics.

Second, work on social influence suggests that immediacy (i.e., physical or psychological distance) affects the likelihood of attitude change. More specifically, the closer a source of information is, or is perceived to be, the more likely it is to exert influence on attitudes and/or behavior (Latané & Wolf, 1981; Sedikides & Jackson, 1990). In light of this work, it is likely that those who perceive science as closer are also more likely to adopt and maintain attitudes in line with publicly communicated scientific evidence.

As mentioned above, the conceptualization of PSYDISC is inspired by the psychological distance dimensions proposed by CLT (Trope & Liberman, 2010). CLT defines psychological distance as the degree to which an object, event, or concept is detached—or “cognitively separated” (Baltatescu, 2014)—from the self in the here and now (Trope & Liberman, 2010). In other words, people perceive objects or concepts as more or less psychologically close (or distant) along four positively associated dimensions (Fiedler et al., 2012)—temporal, spatial, social, and hypothetical distance. Stimuli that are perceived as psychologically distant invite more abstract, simple, and generalized evaluations. Conversely, stimuli that are perceived as psychologically close invite more concrete, detailed, and contextualized evaluations (Trope & Liberman, 2010).

Although CLT is the most prevalent framework for studying psychological distance in recent years, previously mentioned work on social influence (i.e., Latané & Wolf, 1981; Sedikides & Jackson, 1990), as well as more recent work (e.g., Brügger, 2020; Maglio, 2020) point to the fact that psychological distance is a broad concept that can be studied from different theoretical perspectives. As a comprehensive framework for understanding science skepticism across domains, PSYDISC draws from CLT to conceptualize psychological distance to science, but it also builds on other lines of work (i.e., social impact theory; Latané & Wolf, 1981) and applications of psychological distance (i.e., climate change attitudes; Brügger, 2020; Loy & Spence, 2020).

## Overview of Studies

This work investigates the relationship between PSYDISC and science skepticism. The overarching hypothesis is that PSYDISC predicts science skepticism across domains, contributing variance over and above demographic, ideological, and knowledge determinants. To test this hypothesis, we developed and tested a novel scale measuring PSYDISC using samples from two countries. In the pilot study, we tested a preliminary version of the scale, using exploratory factor analysis (EFA) to select items for the final version of the scale.^1^ We tested the factor structure of the final scale in Studies 1 (EFA) and 2 (confirmatory factor analysis; CFA) in the United Kingdom, as well as in the United States (CFA) in Study 3 (preregistered). In Studies 1 and 2, we also tested the scale’s convergent/divergent validity, and its predictive validity for science skepticism. Due to the high similarity between the samples and study aims, we describe Studies 1 and 2 jointly. In Study 4, we demonstrate PSYDISC’s incremental validity in predicting science skepticism over and above three science attitude scales. Finally, in Study 5 (preregistered), a follow-up with participants who took part in Studies 1 or 2, we assessed the predictive validity of PSYDISC beyond self-reported science skepticism, by focusing on COVID-19 vaccination behavior. An overview of sample characteristics for all studies is given in Table 1.

**Table 1.** Purpose of Study and Sample Characteristics Across All Studies.

	**Pilot study** (UK; *N* = 410)	**Study 1** (UK; *N* = 286)	**Study 2** (UK; *N* = 311)	**Study 3** (US; *N* = 271)	**Study 4**(UK; *N* = 352)	**Study 5** (UK; *N*^1^ = 436)
Study aims	EFA test of the preliminary PSYDISC scale through EFA	EFA test of the structure of the final PSYDISC scale, construct, and predictive validity	CFA test of structure of the final PSYDISC scale, construct, and predictive validity	Generalizability of the PSYDISC factor structure through CFA in a different population	Incremental validity of PSYDISC for predicting science skepticism	Prospective predictive validity of PSYDISC for vaccination hesitancy and behavior
Gender %	Women: 67.1	Women: 61.5	Women: 55.9	Women: 59.0	Women: 48.6	Women: 58.7
	Men: 31.7	Men: 38.1	Men: 43.1	Men: 40.2	Men: 49.7	Men: 40.4
	Other/prefer not to say: 1.2	Other/prefer not to say: 0.3	Other/prefer not to say: 1.0	Other/prefer not to say: 0.7	Other/prefer not to say: 1.7	Other/prefer not to say: 0.9
Age (years)	*M =* 33.46	*M =*36.80	*M =* 37.87	*M =* 31.21	*M =* 38.52	*M =* 39.78
	*SD =* 11.57	*SD =* 13.63	*SD =* 13.64	*SD =* 10.80	*SD =* 13.96	*SD =* 13.60
Education (years)	*M =* 16.20	*M =*16.21	*M =* 16.08	*M =* 16.22	*M =* 16.40	*M =* 16.09
	*SD =* 3.28	*SD =* 3.05	*SD =* 3.16	*SD =* 3.14	*SD =* 3.43	*SD =* 3.22
Subjective SES	*M =* 5.27	*M =* 5.56	*M =* 5.33	*M =* 5.39	*M =* 5.20	*M =* 5.39
	*SD =* 1.53	*SD =* 1.49	*SD =*1.59	*SD =*1.58	*SD =* 1.64	*SD =*1.58
Science training %	None: 67.6	None: 72.4	None: 74.3	None: 52.8	None: 69.3	None: 74.5
	University training: 28.5	University training: 26.2	University training: 22.8	University training: 42.4	University training: 23.3	University training: 22.9
	Working scientist: 5.4	Working scientist: 3.1	Working scientist: 5.1	Working scientist: 14.0	Working scientist: 7.5	Working scientist: 4.4
Political ideology	*M =* 4.00	*M =* 4.32	*M =* 4.37	*M =* 3.71	*M =* 4.04	*M =* 4.45
	*SD =* 1.75	*SD =* 1.75	*SD =* 1.76	*SD =* 2.23	*SD =* 1.59	*SD =* 1.79
Religiosity	*M =* 2.36	*M =* 2.07	*M =* 2.30	*M =* 2.75	*M =* 2.13	*M =* 2.23
	*SD =* 1.78	*SD =* 1.50	*SD =* 1.71	*SD =* 1.90	*SD =* 1.63	*SD =* 1.65

*Note.* EFA = exploratory factor analysis; PSYDISC = psychological distance to science; CFA = confirmatory factor analysis; SES = socioeconomic status.

1 Subset of Studies 1 and 2.

## Pilot Study: Item Construction and Selection

### Method

All studies were approved by the first author’s university ethics committee. We obtained informed consent from all participants recruited across all our studies. Participants were paid £2.13 for their participation in the pilot study.

#### Transparency and openness

We report how we determined our sample size, all data exclusions, and all measures in the study. Data, syntax, research materials, and codebooks are available at: https://osf.io/nz5va/ for all studies. Pilot study data were analyzed using IBM SPSS Statistics (Version 27). This study’s design and analysis were not preregistered.

#### Participants and procedure

Although there are no straightforward procedures for calculating a priori power for factor analyses, recent recommendations suggest the minimum sample size to be between 300 and 400 (Goretzko et al., 2019). To account for inattentive participants, 422 Prolific participants residing in the United Kingdom took part in the study. After excluding participants who did not pass both attention checks, our final sample consisted of 410 participants (275 female, two non-binary, three preferred not to say; *M_age_* = 33.46, *SD_age_* = 11.57). On average, our participants had 16.2 (*SD* = 3.28) years of formal education. For a more detailed overview of sample characteristics across all studies, see Table 1 in the main text.

Participants first responded to the PSYDISC scale items in a randomized order. Afterward, they responded to other science attitude and knowledge measures. Finally, participants responded to a set of demographics questions.

#### Measures

##### PSYDISC scale

Based on a previous scale developed as part of an unpublished doctoral dissertation (McPhetres, 2019) and further conceptual refinements by all authors, we developed 34 items to examine in the pilot study. We based item construction around the four dimensions of psychological distance: temporal, spatial, social, and hypothetical. Details on item conceptualization in relation to the four distance dimensions are available in Supplemental Materials A. Ten items assessed the extent to which people perceived science as hypothetical and not applicable in real life (hypothetical distance; e.g., *Science is mostly concerned with speculation that is not useful in real life; Science is too complicated to be useful in real life*). Ten items tapped into perceptions of scientists as different and unapproachable (social distance; e.g., *Scientists are very different from me; It would be difficult for me to meet with a scientist*). Furthermore, seven items gauged the perceptions of the presence of science in one’s local surroundings (spatial distance; e.g., *Scientific research really contributes to my local area* [reverse-coded]; *Very few scientists live or work in my town*). Finally, seven items assessed perceptions of relevance of science for the present (temporal distance; e.g., *Compared to its past achievements, science has become less relevant; Science is mainly focused on issues that are not relevant right now*). All items are shown in Supplemental Materials A (Table S1).

#### Results

**Figure 1.**
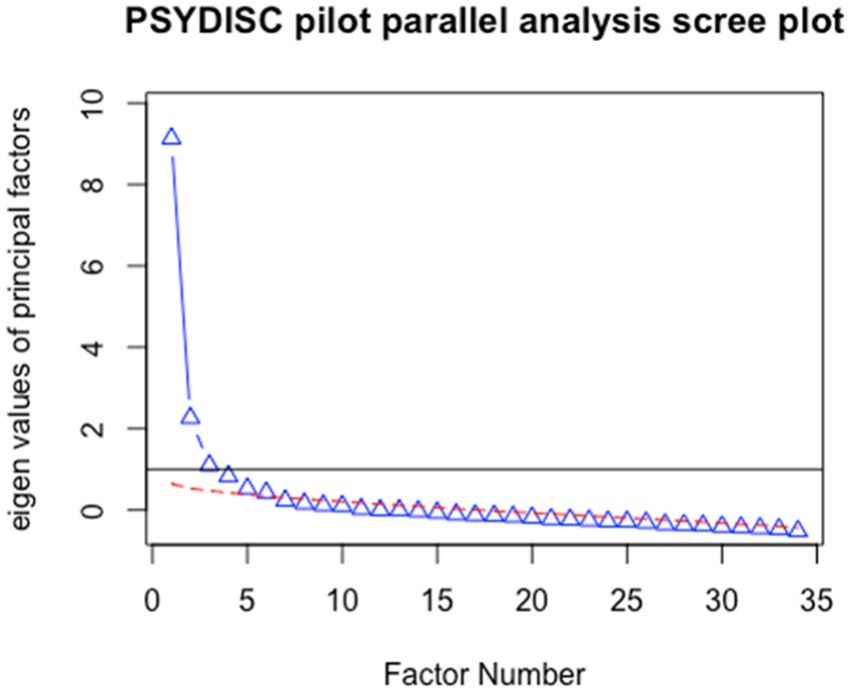
PSYDISC items scree plot; pilot study. *Note.* The dotted line represents eigenvalues of resampled data. PSYDISC = psychological distance to science.

The factor structure of the PSYDISC scale was tested using EFA. We used the principal axis factoring extraction method with a Promax rotation (Kappa = 4). By examining the scree plot (Figure 1), we could justify retaining three, four, five, or six factors, so we ran a parallel analysis (Horn, 1965) using the SPSS raw.par macro (O’Connor, 2000). The parallel analysis (PA) was done based on 1,000 permutations of raw data, which preserves original item distributions. This analysis suggested retaining five factors (when looking at both the mean and the 95% criterion). We examined the five-factor solution first but found that the fifth factor contained only two items with high loadings (>.40), which signals a poorly specified factor (MacCallum et al., 1999). In addition, PA on principal factors (as opposed to components) tends to overestimate the number of factors (Buja & Eyuboglu, 1992). We also inspected the minimum average partial (MAP) and very simple structure (VSS) criteria using the *psych* package in R. The MAP indicated four, while the VSS indicated three factors.

In line with the parallel analysis and the MAP criterion, we proceeded to examine the four-factor solution, which produced four well-defined factors.^2^ The pattern matrix of the full pilot scale is provided in Supplemental Materials A.^3^ As can be seen from Table S1, Factor 1 consisted of social distance items, while Factor 4 consisted of spatial distance items. Factor 2 was most saturated by two temporal items that reflect the view that science used to be more relevant in the past but has since lost significance. The next several items tap into the hypotheticality of science. Factor 3 was comprised of items reflecting views that science is tangible and applicable in the present, which represented a mix of the temporal and hypothetical dimension. Given that we had two clearly defined factors consistent with the theoretical model we used for scale construction, we opted to further conceptually refine the temporal and hypothetical dimensions. Therefore, for subsequent studies, we retained the four highest loading items from Factor 1 and Factor 4.^4^ The temporal dimension was redefined more narrowly in relation to the *future*—as a view that science is oriented toward the more distant future and therefore not that useful for the present moment. Consequently, we refined hypothetical items from Factor 3 to refer to perceiving science as useful, applicable, and accurate, without any temporal references.

## Studies 1 and 2

In Studies 1 and 2, we aimed to test the factor structure (EFA in Study 1 and CFA in Study 2) of the refined PSYDISC scale and investigate its validity. Given that this is the first attempt to gauge distance perceptions to science, we relied on face-valid novel measures to establish convergent construct validity. We did this by estimating correlations of PSYDISC with perceived personal relevance of science, as well as a one-item distance to science slider measure. Furthermore, we aimed to establish divergent validity by estimating correlations with science attitudes, science knowledge and understanding, as well as ideological variables. In addition, we tested the predictive validity of the scale by assessing its contribution to predicting science skepticism across domains over and above previously established predictors.

### Method

Both studies were approved by the first author’s University ethics committee (Study 1: 2020-SP-12934; Study 2: 2021-SP-13190). Participants were paid £2.13 and £2.38 for their participation, respectively, for Studies 1 and 2.

#### Transparency and openness

We report how we determined our sample sizes, all data exclusions, and all measures in both studies. Data were analyzed using IBM SPSS Statistics (Version 27) and R, version 4.1.1 (R Core Team, 2021). The design and analyses of both studies were not preregistered.

#### Participants

For a detailed overview of sample characteristics, see Table 1.

##### Study 1

Three hundred and fourteen Prolific workers residing in the United Kingdom took part in the study. After excluding participants who did not pass both attention checks, our final sample consisted of 286 participants (176 female, one trans woman; *M_age_* = 36.80, *SD_age_* = 13.63). Due to more inattentive participants than expected, this is slightly lower than the 300 minimum recommendations (Goretzko et al., 2019). In terms of the regressions conducted for assessing predictive validity, sensitivity analyses showed that we achieved 90% power to detect an effect of *f*^2^ = .037 in a multiple regression with 12 predictors.

On average, our participants had 16.21 (*SD* = 3.05) years of formal education. Most of the sample identified as atheist (37.8%), agnostic (19.2%), or Catholic (10.1%).

##### Study 2

We recruited 331 U.K. residents on Prolific Academic. After excluding inattentive participants and two suspicious responses (that had the same Prolific IDs), 311 participants made up the final sample. Our sample size was based on a priori power calculations for multiple regressions.^5^ Taking the smallest incremental contribution of the psychological distance to science scale in predicting skepticism in Study 1—6% of additional variance explained in the largest multiple regression (12 predictors), we calculated we needed 296 participants for 95% power to detect the same increase. To account for inattention and data quality, we slightly oversampled. On average, our participants had 16.21 (*SD* = 3.05) years of formal education.

#### Measures

Along with the psychological distance to science scale, to examine the scale’s construct and predictive validity, participants responded to different items tapping into constructs we presumed to be related to psychological distance to science, as well as previous predictors of science skepticism we controlled for in testing predictive validity. Unless otherwise stated, measures described below were included in both Studies 1 and 2 and participants indicated their agreement on a scale from 1 (*strongly disagree*) to 7 (*strongly agree*).

##### Personal relevance of science

We created five items to assess the extent to which people perceive science as relevant to their own lives. The items were the following: “Science is irrelevant to my life,” “Science plays no role in my life,” “Science feels very remote from me,” “Science makes daily life easier” (reverse-coded), and “Science has little to do with me.” This scale was reliable (Study 1: α = .79; Study 2: α = .84) and unidimensional—all items had high loadings on one component in a principal component analysis with Varimax rotation, which explained 56.33% and 63.16% of the variance in Studies 1 and 2, respectively.

##### Global assessment of distance to science

Using a one item slider, we measured the global perception of distance to science. Participants read: “Some concepts can feel distant, while others can feel close to ourselves and our lives. In that regard, how close or distant does science feel to you?” and indicated their response on a slider scale from 0 (*very close to me*) to 100 (*very distant from me*).

##### Faith in science

In Study 1, a five-item, shortened version of the Belief in Science Scale (Farias et al., 2013) obtained from previous studies (Rutjens et al., 2018) was used. The items were as follows: “The scientific method is the only reliable path to knowledge,” “The only real kind of knowledge we can have is scientific knowledge,” “We believe too often in science, and not enough in feelings and faith,” “Science tells us everything there is to know about what reality consists of,” and “Science is the most efficient means of attaining truth.” In Study 2, we used the original 10-item scale from Farias and colleagues (2013). Both versions showed high reliability (Study 1: α = .82; Study 2: α = .88).

##### General science attitudes (Study 1: α = 65; Study 2: α = .60)

To capture more general attitudes toward science, we used six items from previous research (McPhetres & Zuckerman, 2018) that highly resemble science attitudes questions in large-scale public opinion surveys: “Scientific research makes life change too fast” (reverse-coded), “The benefits of scientific research outweigh any possible harms,” “The world is better because of science,” “Science and technology make more opportunities for the next generation,” “Scientists want to make life better,” and “It is not important to know about science in daily life” (reverse-coded).

##### Science interest

To assess participants’ interest in different scientific topics, we asked participants to rate their interest in 30 topics presented in alphabetical order (McPhetres et al., 2021). Fifteen topics were science-related (e.g., discoveries of new animals, robotics), while the other 15 were non-science topics (books, music) on scales ranging from 1 (*not at all interested*) to 7 (*extremely interested*). The topics were averaged into a science interest score (Study 1: α = .93; Study 2: α = .92) and a non-science interest score (Study 1: α = .76; Study 2: α = .76).

##### Science funding support

In Study 2, we asked participants how much science should be funded on a scale from 0 (*as little as possible*) to 100 (*as much as possible*): “In your opinion, how much money should the UK government spend on science?” Similar funding support measures have been used in previous research (Rutjens et al., 2018).

##### Science knowledge

To measure general science knowledge about uncontested facts, we asked participants to indicate whether 13 statements about scientific facts were true or false. The items were adapted from previous research and included questions such as “Electrons are smaller than atoms” (Kahan et al., 2012; Rutjens et al., 2018). The final score was computed as a sum of correct responses on all 13 items.

##### Science understanding

We used a measure of science reasoning skills to assess the level of skills in evaluating scientific findings (Drummond & Fischhoff, 2017a). Participants read short scenarios probing the understanding of basic scientific principles (e.g., ecological validity, randomization, and confounds). After every scenario, participants read a statement about it, for which they needed to determine whether it was true or false. The final score was computed as a sum of correct responses on all 11 items.

##### Science skepticism

We measured science skepticism for climate change (Study 1: α = .83; Study 2: α = .83), vaccination (Study 1: α = .86; Study 2: α = .80), genetically modified (GM) foods (Study 1: α = .90; Study 2: α = .88), and evolution (Study 1: α = .88; Study 2: α = .85), using five-item scales from previous research (Lewandowsky et al., 2013; Lombrozo et al., 2008). In addition to these contested domains, in Study 2 we also measured attitudes toward one novel science domain—genetic editing of human DNA. For this, we used eight items, five of which were highly comparable with items from Lewandowsky and colleagues’ (2013) vaccine and GM food skepticism scales, with the addition of three items tapping into concerns specific for the domain of human genetic editing. This new scale showed good reliability (α = .87) and is included in Supplemental Materials B. After reverse-coding, separate average scores were calculated for each domain.

##### Conspiracy beliefs

We used a single item to measure general proneness to conspiracy beliefs (Lantian et al., 2016). Participants were presented with a short statement about well-known events and asked to indicate whether the statement was true or false on a scale from 1 (*completely false*) to 9 (*completely true*): “I think that the official version of the events given by the authorities very often hides the truth.”

##### Political ideology

We measured political ideology using two items. We asked participants the extent to which they considered themselves left-/right-wing in terms of economic and social issues from 1 (*left-wing/progressive*) to 10 (*right-wing/conservative*). These items were highly correlated (Study 1: *r* = .75; Study 2: *r* = .74) and therefore averaged into one score.

##### Religiosity

Participants reported to what extent they considered themselves religious on a scale from 1 (*not religious at all*) to 7 (*very religious*). In addition, we measured religious orthodoxy with two items (Study 1: *r* = .71; Study 2: *r* = .77; Fontaine et al., 2003; Rutjens & van der Lee, 2020). Participants expressed their agreement with two statements: “God has been defined for once and for all and therefore is immutable” and “Religion is the one thing that gives meaning to life in all its aspects.”

##### Spirituality

We measured the extent to which participants self-identified as spiritual using two items (Study 1: *r* = .85; Study 2: *r* = .88; Rutjens et al., 2018). Participants indicated on a scale from 1 (*not at all*) to 7 (*very much*) whether they considered themselves as spiritual and whether other people consider them as spiritual.

##### Demographics

We asked participants to report their gender, age, religious denomination, years of formal education, subjective social status, and whether they obtained any science training. In Study 1, we also asked participants about their COVID-19 vaccination intentions, which is outside the scope of this article.

### Results

#### Scale structure

##### Study 1, exploratory factor analysis

The EFA was conducted in the same way as in the pilot study—we first determined the number of factors to be retained by conducting parallel analysis (Horn, 1965) on all 17 items of the scale. Both the means and the 95% percentile criterion indicated that four factors should be retained. We then conducted an EFA using the principal axis factoring extraction method and Promax rotation, with the number of factors fixed to 4. The Kaiser–Meyer–Olkin (KMO) measure of sampling adequacy was .84, and the Bartlett’s test of sphericity was significant: χ^2^(136) = 1,927.98, *p* < .001. The four extracted factors explained 16.20%, 13.23%, 11.86%, and 10.87% of variance, respectively. As can be seen in Table 2, all but one of the 17 items had high (>.40) factor loadings on a single factor. Therefore, this item was omitted, resulting in a final scale of 16 items, four items per factor. Internal consistencies of the total scale (α = .85), as well as temporal (α = .86), social (α = .81), hypothetical (α = .77) and spatial (α=.76) subscales﻿ were good. The final scale is also attached in the Appendix.

**Table 2.** PSYDISC Exploratory Factor Analysis Pattern Loadings and Communalities, Study 1.

	Factor loading				*h* ^2^
PSYDISC item wording	1	2	3	4	
Factor 1: Temporal distance					
Most of today’s science is concerned with solving problems of the distant future.	.912				.751
Science is mainly focused on the distant future.	.876				.755
Scientists spend most of their time working on issues of the distant future.	.860				.752
We will see the impact of science more in the distant future than we do in the present.	.533				.328
Factor 2: Social distance					
The prospect of working as a scientist seems beyond my reach.		.830			.586
I rarely interact with scientists in real life.		.753			.550
Scientists are very different from me.		.642			.514
It would be difficult for me to meet with a scientist.		.562			.487
Factor 3: Hypothetical distance					
Scientific knowledge is a reliable way to solve important issues.			.765		.615
Science provides accurate information about the world we live in.			.742		.533
We can rely on science to deliver results that can be implemented in real life.			.679		.468
I can see the effects of science, whether positive or negative, on the world.			.449		.338
For science to solve important issues, patience is required.^a^			.345		.196
Factor 4: Spatial distance					
Science and scientific research play a big role in my local area.				−.848	.635
Scientific research really contributes to my local area.				−.622	.447
People from my local area don’t become scientists.				.551	.502
Very few scientists live or work in my town.				.483	.409

*Note.* The extraction method was principal axis factoring with an oblique (Promax with Kaiser Normalization) rotation. Factor loadings below .10 are not shown.

a Item omitted due to low primary loading and communality.

##### Study 2—confirmatory factor analysis

To confirm the factor structure obtained using EFA in Study 1, we conducted a CFA on the 16 PSYDISC items. We tested a model comprising of four first-order latent factors that correspond to the four factors obtained through EFA in Study 1 as well as one second-order factor, representing overall PSYDISC and thus relating to the four first-order latent factors. Internal consistencies of the total scale (α = .86), as well as temporal (α = .87), social (α = .83), hypothetical (α = .79) and spatial (α = .77) subscales﻿ were good. Due to non-normality of some items, we used MLM estimation in the *lavaan* (Rosseel, 2012; version 0.6-5) package in R. For the same reason, we used robust fit indices to estimate model fit. More specifically, we used robust versions of the Comparative Fit Index (CFI), Tucker–Lewis Index (TLI), the Standardized Root Mean Square Residual (SRMR), as well as the Root Mean Square Error of Approximation (RMSEA). We did not consider the Chi-square test, as it is an unreliable fit index due to its sensitivity to sample size—it usually yields significant results (indicating poor model fit) for sample sizes larger than 200 (Steiger, 2007).

CFA revealed that this four-factor model was a good fit to the data, robust χ^2^(99) = 194.25, *p* < .001; robust CFI = .96, TLI = .95, RMSEA = .06 [90% CI = .044, .069], SRMR = .07. This fit was achieved after adding one parameter for correlated errors of manifest variables—namely, between two items from the spatial dimension. We deemed this acceptable as the items were from the same subscale, indicating there is more similarity between some indicators of the same dimension (likely due to items being reverse-coded), as opposed to cross-dimension covariance which would signal poorly specified factors.

#### Construct validity across Studies 1 and 2

To facilitate comparisons between construct validity tests in Studies 1 and 2, we present these results jointly.

##### Intercorrelations and convergent validity

As can be seen in Table 3, across both studies, the PSYDISC scale subdimensions correlated positively (|*r*|s from .21 to .51, *p*s <.01). The total score, as well as all subscale scores, had moderate-to-high positive correlations with perceived personal relevance of science. Consistent with our conceptualization, this suggests that PSYDISC relates to the overall perception of relevance of science in one’s own life. As for the one-item distance slider, PSYDISC was also—as was to be expected—positively related to it.

**Table 3.** PSYDISC Intercorrelations, Personal Relevance of Science and Distance Slider Correlations, Studies 1 and 2.

	Social	Spatial	Temporal	Hypothetical	Personal relevance of science	Distance slider
Study 1						
PSYDISC	.812*** [.764, .851]	.743*** [.684, .793]	.707*** [.641, .762]	.495*** [.397, .579]	−.648*** [.581, .710]	.469*** [.354, .574]
Social	—	.493*** [.395, .585]	.355*** [.242, .456]	.270*** [.156, .370]	−.571*** [−.654, −.482]	.472*** [.359, .575]
Spatial		—	.319*** [.212, .417]	.252*** [.135 .358]	−.403*** [−.508, −.296]	.260*** [.156, .365]
Temporal			—	.210*** [.089, .314]	−.382*** [−.473, −.288]	.227*** [.101, .348]
Hypothetical				—	−.504*** [−.587, −.420]	.386*** [.276, .494]
*M* (*SD*)	4.20 (1.44)	3.68 (1.12)	3.54 (1.28)	1.90 (.67)	5.69 (.93)	37.94 (24.50)
Study 2						
PSYDISC	.803*** [.760, .841]	.757*** [.702, .802]	.727*** [.679, .774]	.528*** [.443, .606]	−.687*** [−.739, −.626]	.529*** [.437, .609]
Social	—	.508*** [.402, .603]	.370*** [.268, .467]	.217*** [.109, .317]	−.585*** [−.652, −.512]	.565*** [.472, .642]
Spatial		—	.350*** [.242, .451]	.308*** [.193, .413]	−.477*** [−.563, −.383]	.358*** [.251, .453]
Temporal			—	.301*** [.199, .397]	−.401*** [−.485, −.313]	.239*** [.123, .342]
Hypothetical				—	−.539*** [−.621, −.441]	.312*** [.202, .404]
*M* (*SD*)	4.25 (1.52)	3.54 (1.12)	3.56 (1.29)	1.97 (.73)	5.58 (1.04)	43.54 (25.37)

*Note.* Pearson’s *r* with 95% bootstrapped BCa CIs [L, U]. Study 1 *N =* 285, Study 2 *N* = 310. PSYDISC = psychological distance to science; CI = confidence interval.

***p < .001.

##### Divergent construct validity

Next, we examined zero-order correlations with variables we expected to moderately correlate with PSYDISC. Overall, these correlations are highly comparable across Studies 1 and 2, as well as in the same direction across PSYDISC dimensions. As shown in Table 4, general science attitudes were consistently negatively related to PSYDISC, while Faith in Science was negatively correlated with overall PSYDISC, as well as social and hypothetical distance.

**Table 4.** PSYDISC Correlations With Science Attitudes, Tests, Interests, and Funding, Studies 1 and 2.

	Faith in Science	General sci. att.	Science know.	Science und.	Science interest	Non-science interest	Science funding
Study 1							
PSYDISC	−.086** [−.326, −.046]	−.441*** [−.533, −.343]	−.293*** [−.390, −.186]	−.377*** [−.466, −.277]	−.264*** [−.369, −.144]	.000 [−.128, .128]	—
Social	−.160** [−.279, −.035]	−.276*** [−.374, −.170]	−.250*** [−.348, −.145]	−.358*** [−.446, −.266]	−.323*** [−.428, −.210]	.021 [−.099, .139]	
Spatial	−.061 [−.199, .079]	−.308*** [−.409, −.201]	−.108 [−.217, .006]	−.148* [−.252, −.036]	−.180** [−.286, −.074]	−.138* [−.253, −.018]	
Temporal	.007 [−.123, .137]	−.241*** [−.361, −.117]	−.267*** [−.380, −.146]	−.336*** [−.434, −.225]	−.020 [−.135, .099]	.117* [−.009, .248]	
Hypothetical	−.464*** [−.563, −.351]	−.563*** [−.636, −.479]	−.188** [−.309, −.066]	−.164** [−.274, −.043]	−.240*** [−.365, −.130]	−.040 [−.163, .073]	
*M* (*SD*)	4.47 (1.18)	5.47 (.78)	10.16 (1.59)	7.17 (2.37)	4.35 (1.15)	4.34 (.79)	
Study 2							
PSYDISC	−.225** [−.324, −.119]	−.429*** [−.513, −.345]	−.318*** [−.407, −.222]	−.360*** [−.457, −.258]	−.300*** [−.402, −.194]	.040 [−.075, .150]	−.326** [−.426, −.220]
Social	−.185** [−.283, −.086]	−.240** [−.341, −.128]	−.282*** [−.378, −.187]	−.320*** [−.415, −.218]	−.334*** [−.428, −.236]	.058 [−.062, .169]	−.164** [−.272, −.052]
Spatial	−.096 [−.211, .018]	−.266*** [−.368, −.161]	−.100 [−.208, .003]	−.202*** [−.320, −.087]	−.208*** [−.318, −.096]	−.070 [−.180, .037]	−.194*** [−.306, −.079]
Temporal	−.013 [−.134, .111]	−.291*** [−.380, −.196]	−.312*** [−.412, −.215]	−.313** [−.410, −.217]	−.056 [−.160, .005]	.153** [.035, −.216]	−.235*** [−.333, −.132]
Hypothetical	−.488*** [−.575, −.395]	−.571*** [−.644, −.495]	−.183** [−.289, −.076]	−.145* [−.255, −.036]	−.282*** [−.392, −.161]	−.101 [−.003, .070]	−.458*** [−.560, −.359]
*M* (*SD*)	4.75 (1.01)	5.40 (.73)	9.89 (1.50)	6.83 (2.45)	4.35 (1.11)	4.34 (.80)	73.68 (17.59)

*Note.* Pearson’s *r* with 95% bootstrapped BCa CIs [L, U]. Study 1 *N* = 286; Study 2 *N* = 311. PSYDISC = psychological distance to science; General sci. att. = general science attitudes; Science know. = science knowledge; Science und. = science understanding; CI = confidence interval.

* *p* < .05. **p < .01. ***p < .001.

Furthermore, PSYDISC (except for the spatial subscale) was negatively correlated with objective tests of factual science knowledge, as well as understanding of the scientific process. Looking at self-reported interests, PSYDISC was predominantly negatively related to interest in science-related topics, as opposed to non-science-related interest, for which the correlations were nonsignificant. The only exception to this was spatial distance in Study 1 being weakly negatively related to both science and non-science interests. Finally, support for national science funding, measured in Study 2, was consistently negatively related to PSYDISC.

In addition to science-related attitudes, interests, knowledge, and support, we also inspected how PSYDISC correlates with ideological variables previously found to determine science attitudes. The results were in line with previous findings, with political conservatism (|*r*|s from .18 to .36, *p*s <.01) and conspiracy beliefs (|*r*|s from .14 to .26, *p*s <.05) being the most consistent negative correlates of PSYDISC (Supplemental Materials C, Table S2).

##### Predictive validity

After examining construct validity, we turned to testing PSYDISC’s predictive validity, which was central to our aim of constructing a scale that predicts science skepticism across domains. In Tables 5 and 6, we show the results from two sets of regressions—the first with the contribution of the total PSYDISC score and the second with its subscales—to predicting science skepticism across Studies 1 and 2. More specifically, we entered age, gender, education, religiosity, spirituality, conspiracy beliefs, political ideology, science knowledge, and science understanding in Step 1, and PSYDISC in Step 2. First, PSYDISC consistently positively predicted skepticism across domains in both studies, confirming our overarching hypothesis. Second, when scrutinizing the subscales, hypothetical distance was the most consistent positive predictor of skepticism across domains. However, other dimensions also contributed to skepticism—temporal distance positively predicted climate change and evolution skepticism, while social distance predicted GM food and genetic editing skepticism. Spatial distance had the lowest contribution, as it marginally predicted only vaccination skepticism in Study 1. Complete results of these regressions, including (Tables S3–S12) and excluding (Tables S13 and S14) covariates, are available in Supplemental Materials D.

**Table 5.** PSYDISC Regression Coefficients for Multiple Regressions Predicting Science Skepticism, Studies 1 and 2.

	Climate change	Vaccination	Evolution	GM foods	Genetic editing
Study 1					
*B* (*SE*)	.23 (.08)	.35 (.07)	.35 (.07)	.50 (.09)	/
*p*	< .001	< .001	< .001	< .001	
*Part r.* [95% CI]	.18 [.07, .30]	.28 [.17, .37]	.29 [.18, .38]	.33 [.22, .43]	
Study 2					
*B* (*SE*)	.34 (.07)	.32 (.07)	.36 (.06)	.22 (.09)	.36 (.07)
*P*	<.001	< .001	< .001	<.001	< .001
*Part r* [95% CI]	.29 [.18, .41]	.27 [.16, .38]	.32 [.21, .43]	.14 [.02, .26]	.28 [.13, .41]

*Note.* Part. *r* = partial correlation coefficient with bootstrapped BCa 95% confidence intervals. Study 1 *N* = 285, Study 2 *N* = 307 due to listwise omission of incomplete cases. We controlled for age, gender, education, religiosity, spirituality, conspiracy belief, political ideology, science knowledge, and science understanding. PSYDISC = psychological distance to science; GM = genetically modified; CI = confidence interval.

**Table 6.** PSYDISC Regression Coefficients for Multiple Regressions Predicting Science Skepticism, Studies 1 and 2.

	Hypothetical			Social			Temporal			Spatial		
	*B* (*SE*)	Part *r*. [*95% CI*]	*p*	*B* (*SE*)	Part *r*. [*95% CI*]	*p*	*B* (*SE*)	Part *r*. [*95% CI*]	*p*	*B* (*SE*)	Part *r*. [*95% CI*]	*p*
Climate change												
Study 1	.37 (.08)	.26 [.12, .41]	< .001	.01 (.05)	.01 [−.10, .12]	.827	.07 (.05)	.09 [−.04, .23]	.136	.00 (.05)	00 [−.14, .13]	.978
Study 2	36 (.07)	.28 [.17, .40]	< .001	.01 (.04)	.01 [−.10, .13]	.863	.10 (.04)	.13 [.01, .25]	.029	.07 (.05)	.07 [−.05, .21]	.199
Vaccination												
Study 1	.46 (.08)	.34 [.21, .45]	< .001	−.02 (.04)	−.03 [−.15, .07]	.828	.08 (.05)	.11 [−.02, .22]	.074	.10 (.05)	.12 [−.02, .25]	.044
Study 2	.48 (.07)	.38 [.24, .48]	< .001	.04 (.04)	.05 [−.05, .15]	.351	.03 (.04)	.04 [−.08, .16]	.524	.02 (.05)	.03 [−.08, .13]	.653
Evolution												
Study 1	42 (.08)	.32 [.18, .45]	< .001	.02 (.04)	.03 [−.09, .13]	.640	.09 (.04)	.12 [.01, .23]	.040	.06 (.05)	.07 [−.06, .20]	.261
Study 2	.36 (.07)	.31 [.19, .42]	< .001	.06 (.04)	.11 [−.02, .23]	.071	.11 (.04)	.15 [.02, .29]	.009	.00 (.05)	−.01 [−.13, .11]	.924
GM foods												
Study 1	.41 (.09)	.26 [.15, .36]	< .001	12 (.05)	.14 [−.02, .26]	.017	.04 (.05)	.05 [−.07, .16]	.422	.10 (.06)	.10 [−.03, .24]	.105
Study 2	.29 (.09)	.18 [.06, .27]	.002	.11 (.05)	.12 [.00, .27]	.036	.02 (.06)	.02 [−.11, .15]	.766	−.08 (.07)	−.07 [−.18, .06]	.227
Genetic editing												
Study 2	.25 (.08)	.18 [.07, .29]	.002	.10 (.04)	.13 [.01, .27]	.021	.03 (.05)	.04 [−.08, .18]	.480	.06 (.06)	.04 [−.08, .18]	.270

*Note.* Part. *r* = partial correlation coefficient with bootstrapped BCa 95% confidence intervals. Study 1 *N* = 285, Study 2 *N* = 307 due to listwise omission of incomplete cases. We controlled for age, gender, education, religiosity, spirituality, conspiracy belief, political ideology, science knowledge, and science understanding. PSYDISC = psychological distance to science; CI = confidence interval; GM = genetically modified.

### Discussion

Taken together, the results of Studies 1 and 2 point to good reliability and construct validity of the PSYDISC scale. First, PSYDISC was consistently related to perceiving science as more personally relevant, and closer on a general distance slider, demonstrating convergent validity. Second, PSYDISC had low-to-moderate negative correlations with Faith in Science, positive science attitudes, science knowledge, science understanding, interest in science topics, as well as support for national science funding, thus showing divergent validity. Most importantly, we found that the PSYDISC scale has additional explanatory power in predicting skepticism across all tested science domains, over and above previously established predictors. Hypothetical distance was a consistent positive predictor across domains and studies, while temporal and social distance also played a role in some domains—temporal for climate change and evolution, social for GM foods and genetic editing in humans. Since both studies were run in the United Kingdom, the question of the replicability and generalizability of the factor structure to other countries remained. Therefore, in Study 3, we proceeded to test the factor structure of the scale in a different country.

## Study 3

To test the generalizability of the factor structure of our scale beyond the context of the United Kingdom, we ran a preregistered CFA study in the United States. The preregistration can be found here: https://osf.io/rw6mz.^6^ We tested the same model as in Study 2 using confirmatory factor analysis—four latent factors corresponding to the four distance dimensions, as well as a higher-order general PSYDISC factor.

### Methods

The study was approved by the first authors’ University ethics committee (2021-SP-13584), and the respondents received £0.63 for their participation.

#### Transparency and openness

We report how we determined our sample size, all data exclusions, and all measures. Data were analyzed using IBM SPSS Statistics (Version 27), as well as R, version 4.1.1 (R Core Team, 2021).

#### Participants

In total, 301 U.S. residents recruited through Prolific Academic completed the study. After excluding those who did not pass the attention check, were flagged as potential bots by Qualtrics—the survey hosting platform—or had duplicate location data, 271 participants remained. This was slightly over the preregistered 252 participants we determined were needed to confirm the factor structure (using RMSEA-based power calculations; Jak et al., 2020); therefore, this study was well-powered (over 95%). Sample characteristics are provided in Table 1.

#### Measures

Besides the psychological distance to science scale (total score α = .84; hypothetical α = .79; social α = .81; temporal α = .86; spatial α = .79), we also measured the global perception of distance to science with a one slider question, as well as personal relevance of science (α = .82) in the identical manner as in Studies 1 and 2. We also asked participants to indicate their political ideology (*r* = .89) and religiosity, equivalent to questions in Studies 1 and 2. In addition, we obtained demographic information on state and place of residence, gender, age, religious denomination, years of formal education, subjective social status, and science training.

### Results and Discussion

The parameters for the CFA were identical to those used in Study 2, as was the model we tested—four first-order latent factors, corresponding to the four distance dimensions and a higher-order general distance factor, with a correlated residual variance between two spatial distance items. Due to nonnormality of some items, we used MLM estimation in the *lavaan* (Rosseel, 2012; version 0.6-9) package in R (version 4.1.1). For the same reason, we again used robust fit indices to estimate model fit. CFA revealed that the four-factor model provided good fit for the data, χ^2^(99) = 143.73, *p* = .002, CFI = .97, TLI = .96, RMSEA = .05 [90% CI = .03, .06], SRMR = .06, indicating that the PSYDISC scale structure generalizes to the U.S. population.

Furthermore, we tested the measurement invariance of our scale across the United Kingdom (sample from Study 2) and the United States (sample from this study). Results demonstrated that our scale had configural, metric, and scalar equivalence across the two samples, indicating that the PSYDISC scale measures an equivalent construct across both countries and thus allowing for comparisons in PSYDISC scores to be made between them. A detailed description of the analysis and the results can be found in Supplemental Materials E (Table S15).

Finally, we tested PSYDISC intercorrelations, zero-order correlations with personal relevance of science and the psychological distance to science slider, as well as basic ideological variables (religiosity and political ideology). These results were in line with findings from Studies 1 and 2 and are provided in Supplemental Materials E (Table S16).

## Study 4

As an additional test in establishing PSYDISC’s validity for predicting science skepticism, we investigated its incremental validity in predicting science skepticism beyond several existing science attitude scales. Scales measuring general science evaluations, such as Faith in Science (Farias et al., 2013; Rutjens et al., 2018), Credibility of Science (CoS; Hartman et al., 2017), and the Negative Perceptions of Science Scale (NPSS; Morgan et al., 2018), have been utilized to predict science skepticism. However, we expected that PSYDISC, measuring fine-grained perceptions of distance to science, would contribute additional variance in predicting skepticism, beyond above-mentioned scales that tap into either evaluations of the epistemic value of science (Faith in Science) or largely negative attitudes toward science (NPSS and CoS).

### Methods

The study was approved by the authors’ university ethics committee (2022-SP-15349), and respondents received £1.07 for participation.

#### Transparency and openness

We report how we determined our sample size, all data exclusions, and all measures. Data were analyzed using IBM SPSS Statistics (Version 27). This study was not preregistered.

#### Participants

In total, 368 U.K. residents were recruited through Prolific Academic. After excluding those who did not pass the attention checks and/or were flagged as potential bots by Qualtrics—the survey hosting platform, 351 (48.7% female) participants remained in the final sample. This provided us with 95% power to detect an increase in explained variance as small as *f*^2^ = .037. Participants’ demographic characteristics are provided in Table 1.

#### Measures

PSYDISC (α = .87) was measured identically to Studies 2 and 3, while Faith in Science (α = .83; Rutjens et al., 2018) was measured as in Study 1, with the 5-item shortened version. Science skepticism in the domains of climate change (α = .87), vaccination (α = .87), evolution (α = .88) and genetically modified foods (α = .88), was measured identically to Studies 1 and 2.

##### Credibility of Science (CoS; α = .90)

CoS (Hartman et al., 2017) was measured using six items (e.g., “People trust scientists a lot more than they should”) answered on a 7-point scale (1 = *strongly disagree*; 7 = *strongly agree*).

##### Negative perceptions of Science Scale (NPSS; α = .90)

The 20-item NPSS (Morgan et al., 2018) inventory was used to tap into negative science attitudes (e.g., “Science produces many contradictory findings”). Participants indicated their agreement with each statement on a scale from 1 (*strongly disagree*) to 5 (*strongly agree*).

### Results

PSYDISC correlated expectedly to science attitude scales. Echoing Study 1 and 2 results, PSYDISC was weakly negatively related to Faith in Science (*r* = .15, *p* = .003; 95% CI [−.26, −.05]). Furthermore, PSYDISC was moderately negatively related to CoS (*r* = −.53, *p* < .001, 95% CI [−.61, −.44]) and moderately positively related to NPSS (*r* = .53, *p* < .001, 95% CI [.45, .60]). Interestingly, NPSS and CoS were related very strongly (*r* = −.78, *p* < .001, 95% CI [−.82, −.73]), indicating substantial overlap between these two constructs.

To gauge the incremental validity of PSYDISC in predicting science skepticism, we conducted four hierarchical linear regressions, with climate change, vaccination, evolution, and genetically modified foods skepticism as outcomes. We entered age, gender, Faith in Science, and NPSS in Step 1,^7^ and added PSYDISC in Step 2.

As shown in Table 7, PSYDISC consistently contributed additional variance (ranging from 2% to 5%) to skepticism across domains. Therefore, this study demonstrates PSYDISC has additional value in accounting for science skepticism over and beyond valenced science attitudes and epistemic evaluations of science.

**Table 7.** Incremental Validity of PSYDISC Over and Beyond Faith in Science and NPSS, Study 4.

Outcome	Model 1			Model 2			Δ*R*^2^
	*B* (*SE*)	Part *r*. [95% CI]	*p*	*B* (*SE*)	Part *r*. [95% CI]	*p*	
Climate change							
Sci. faith	−.10 (.05)	−.10 [−.22, .02]	.064	−.14 (.05)	−.14 [−.26, −.02]	.008	
NPSS	.72 (.10)	.36 [.26, .45]	< .001	.43 (.12)	.20 [.10, .29]	< .001	
PSYDISC	—	—	—	.33 (.07)	.25 [.14, .36]	< .001	5%
Vaccination							
Sci. faith	−.11 (.05)	−.12 [−.23, −.01]	.023	−.14 (.05)	−.16 [−.26, −.05]	.003	
NPSS	.95 (.09)	.48 [.38, .57]	< .001	.72 (.11)	.34 [.24, .44]	< .001	
PSYDISC	—	—	—	.26 (.06)	.21 [.12, .31]	< .001	3%
Evolution							
Sci. faith	−.13 (.05)	−.15 [−.27, −.02]	.007	−.15 (.05)	−.17 [−.29, −.05]	.002	
NPSS	.77 (.09)	.41 [.30, .51]	< .001	.61 (.11)	.30 [.18, .41]	< .001	
PSYDISC	—	—	—	.18 (.06)	.15 [.06, .25]	.005	2%
GM foods							
Sci. faith	−.13 (.06)	−.12 [−.23, −.01]	.031	−.17 (.06)	−.15 [−.26, −.04]	.005	
NPSS	.62 (.11)	.29 [.17, .39]	< .001	.34 (.13)	.14 [.04, .25]	.009	
PSYDISC	—	—	—	.32 (.08)	.22 [.11, .33]	< .001	4%

*Note. N* = 345 due to listwise omission of incomplete cases. PSYDISC = Psychological Distance to Science; NPSS = Negative Perceptions of Science Scale; Sci. faith = Faith in Science; Part. *r* = Partial *r*.; CI = confidence interval; GM = genetically modified.

## Study 5

Having established that the PSYDISC scale predicts science skepticism across domains, we wanted to examine whether the predictive validity of PSYDISC extends beyond self-reported science skepticism to its downstream, behavioral consequences. Vaccination skepticism is a key barrier for vaccine uptake (e.g., Betsch et al., 2015; El-Mohandes et al., 2021), which has far-reaching consequences for combatting infectious diseases such as COVID-19 or measles. Therefore, in Study 5, we tested whether PSYDISC predicts COVID-19 vaccination hesitancy and behavior. We conducted a follow-up study with participants from Studies 1 and 2, asking about their vaccination status and how much they hesitated in case they did receive the vaccine. We hypothesized that, controlling for demographics, ideological variables, and science knowledge and understanding, PSYDISC would predict (a) a lower likelihood of being fully vaccinated against COVID-19 and (b) higher hesitancy when making the decision to get vaccinated.

### Methods

The study was approved by the authors’ university ethics committee (2021-SP-14018), and respondents received £0.25 for participation.

#### Transparency and openness

We report how we determined our sample size, all data exclusions, and all measures. Data were analyzed using IBM SPSS Statistics (Version 27). The design, hypotheses and analyses were preregistered: https://osf.io/qbe76.

#### Participants

All participants who took part in Study 1 or 2 were eligible to participate. Out of that pool, 553 participants were still active on Prolific at the time of study launch. Given that by that time (November 2021), most of the U.K. population had been vaccinated, we aimed to collect data from all eligible participants, to obtain as many unvaccinated individuals in our sample as possible. Data collection was open for 3 weeks. After excluding those who did not pass attention checks in the first studies, or were flagged as potential bots by Qualtrics, the survey hosting platform, 436 participants were left for the final analyses. As this sample was a subset from samples recruited in Studies 1 and 2, sample characteristics were highly similar (see Table 1). The sample was predominantly fully vaccinated (86.9%), with 10.6% of participants who did not receive any dose of a COVID-19 vaccine.

#### Measures

All predictors and demographics were measured in Studies 1 and 2 and were therefore not assessed again. In addition to two dependent variables described below, we measured vaccination status of children (if the participant was a parent),^8^ as well as reasons for vaccination (for exploratory purposes).

##### Vaccination status

After reminding participants of survey anonymity in order to encourage honest responses, we asked them about their vaccination behavior as follows: “We’d like to know which vaccination status applies to you. Please choose one of the following.” Participants could choose between the following options: “I am fully vaccinated against COVID-19.”; “I am partly vaccinated: I’ve had one shot of a two-dose COVID-19 vaccine.”; “I haven’t received any COVID-19 vaccine doses even though I am eligible.”; or “I am not eligible to receive the COVID-19 vaccine due to underlying health conditions.” We recoded the responses to reflect fully vaccinated status; participants were thus grouped as either fully vaccinated, or not fully vaccinated. We planned to exclude participants with underlying health conditions from the analysis, but there were none in our sample.

##### Vaccine hesitancy

Subsequently, we asked all vaccinated individuals to indicate their level of hesitancy to receive the COVID-19 vaccine with the following item: To what extent did you hesitate when deciding whether to get the COVID-19 vaccine?” Participants responded on a 7-point scale (1 = *not at all*; 7 = *a great deal*).

### Results and Discussion

#### Vaccination hesitancy

First, we investigated whether the degree of hesitancy among the fully vaccinated individuals in the study was predicted by PSYDISC. Due to the severe skewedness of vaccination hesitancy (over 60% of participants reported not hesitating at all), we could not conduct a stepwise linear regression as planned in the preregistration, due to the non-normality of residuals. We instead ran an ordinal regression with the same preregistered predictors. Results showed that PSYDISC, controlling for demographics, ideological and knowledge predictors, positively predicted COVID-19 vaccination hesitancy within the vaccinated population, *B*(*SE*) = .54 (.16), 95% CI [.23, .85], *Wald* = 11.81, *p* < . 001. Looking at PSYDISC subscales, individuals who perceived more social distance to science reported hesitating more when making the decision to get vaccinated. Full regression results are available in Supplemental Materials F (Table S17).

#### Vaccination status

**Figure 2.**
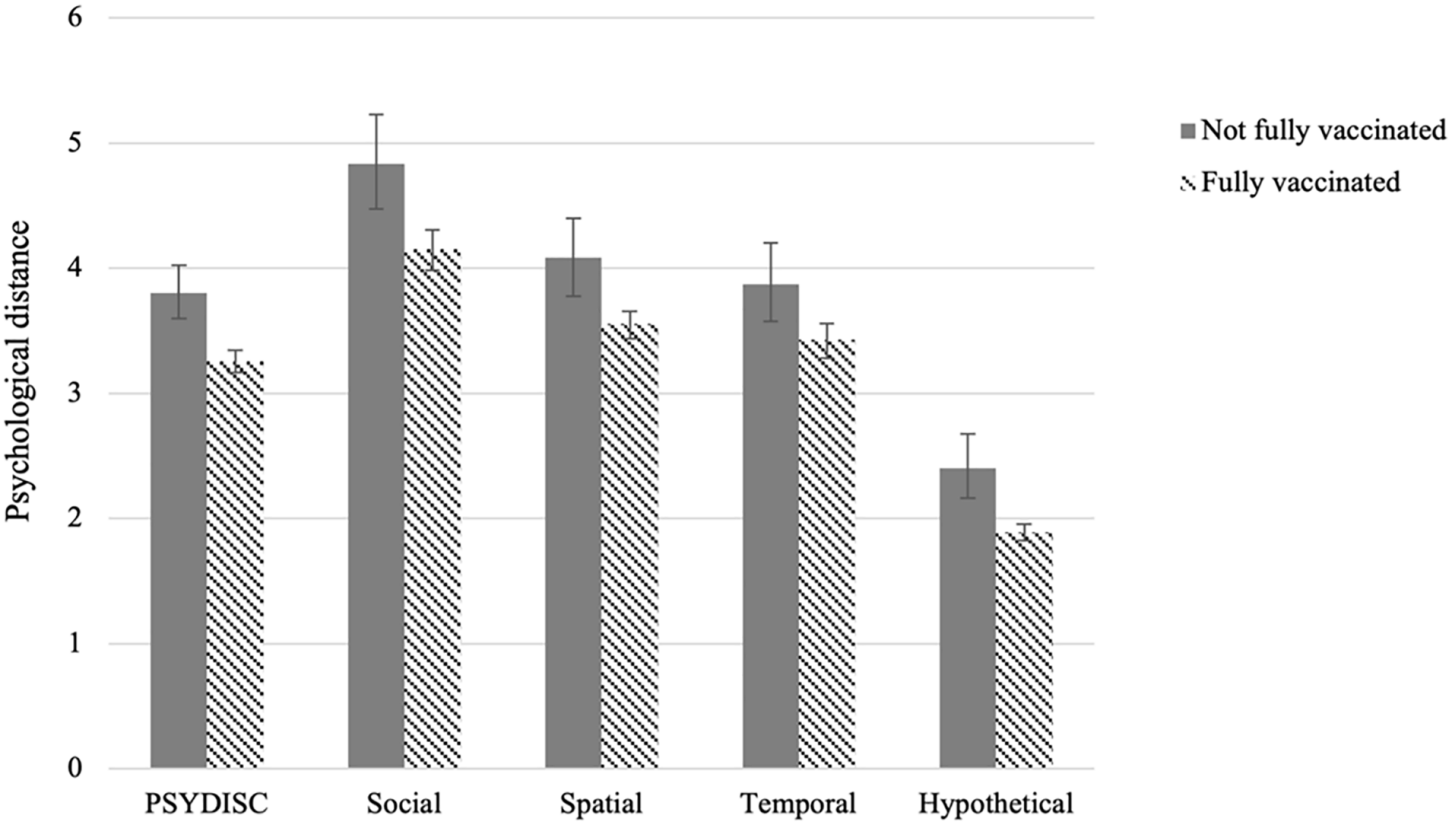
PSYDISC means for vaccinated and unvaccinated individuals, Study 5. *Note.* Error bars represent 95% BCa CIs. PSYDISC = psychological distance to science; CI = confidence interval.

In Figure 2, we present an overview of the differences in PSYDISC scores for vaccinated and unvaccinated individuals. PSYDISC was higher in the unvaccinated, compared with the vaccinated group. All differences were statistically significant on the *p* < .05 level or lower.

To account for other predictors, we then performed the preregistered analysis—a logistic regression with vaccination status as the outcome. Controlling for demographics, ideological beliefs, as well as science knowledge and understanding, PSYDISC—measured 8 or 10 months prior—predicted being fully vaccinated, *B*(*SE*) = −.59 (.22), OR [95% CI] = .55 [.36, .85]; *p* = .007. Of the subscales, hypothetical distance was a negative predictor of being fully vaccinated. Complete regression results are available in Supplementary Materials F (Table S18).

It is notable that different aspects of PSYDISC are important for different COVID-19 vaccination-related outcomes—vaccination uptake was predicted by hypothetical distance, while vaccination hesitancy was predicted by social distance to science. However, these outcomes differ in important ways. While vaccination uptake reflects actual behavior, hesitancy taps into subjective post hoc reasoning about an already made decision. Our results suggest that perceiving science as an applicable and useful endeavor is a prerequisite for making the decision to get vaccinated. However, those who perceive scientists as unrelatable and unapproachable might feel more ambivalent about their decision to do so.

In sum, Study 5 provided evidence for behavioral consequences of PSYDISC. These results demonstrate that the predictive power of PSYDISC extends beyond self-reported science skepticism into skepticism-related behavioral outcomes.

## General Discussion

The ongoing COVID-19 pandemic has brought about disbelief in, and noncompliance with scientific advice, once more making it unequivocally clear that science skepticism has tangible societal consequences. However, the detrimental effects of science skepticism extend widely beyond the pandemic. Given the urgency of action on climate change (Intergovernmental Panel on Climate Change, 2021), the need to be better prepared for future pandemics, and to maintain progress in evolution-based biomedical sciences, it is imperative to deepen our understanding of the psychological roots of science skepticism. In the present research, we introduced and tested Psychological Distance to Science (PSYDISC). Aimed at understanding and systematically predicting science skepticism across domains, this theoretically informed construct refers to perceptions of science in terms of its tangibility and relevance for the individual, reflected in the four psychological distance dimensions (spatial, temporal, social, and hypothetical distance).

Across three studies and two countries (the UK and the US), the PSYDISC scale showed the expected factor structure (four factors corresponding to four distance dimensions and a higher-order general PSYDISC factor). In addition, the scale demonstrated good construct validity through expected correlations with science knowledge variables, science attitude and interest scales, as well as ideological variables. Crucially, the scale showed excellent predictive validity for science skepticism. More specifically, after accounting for demographics and various ideological and knowledge predictors, PSYDISC predicted significant additional variance for climate change, vaccination, evolution, GM foods, and genetic editing skepticism. This means that PSYDISC is an important predictor of science skepticism across all tested science domains. Furthermore, Study 4 demonstrated that the predictive value of PSYDISC holds over and above general valenced science attitudes, captured by Faith in Science and Negative Perceptions of Science Scale.

In addition to the predictive validity of PSYDISC for science skepticism, we also tested whether the scale’s predictive power extends into behavioral outcomes related to science skepticism. To achieve this, we conducted a preregistered follow-up study by recruiting participants from Studies 1 and 2. We found that PSYDISC prospectively predicted COVID-19 vaccination status, as well as subjective vaccination hesitancy. More specifically, higher hypothetical distance predicted lower chances of being fully vaccinated, while social distance predicted more hesitancy when making the decision to get vaccinated or not.

### Hypothetical Distance to Science Predicts Skepticism Across Domains

In terms of the predictive power of the individual psychological distance dimensions, hypothetical distance was a common predictor of science skepticism across all domains, pointing to the importance of the perceived hypothetical nature of science in shaping attitudes across various publicly contested science domains. Although indirectly related experimental work has shown that different types of uncertainty about specific findings can have diverse (and often inconsistent) effects on science attitudes (Gustafson & Rice, 2020; van der Bles et al., 2019), our results suggest that broader perceptions of the applicability and usefulness of science are important in shaping science skepticism across domains.

### The Roles of Temporal and Social Distance to Science Vary Per Domain

The predictive power of other distance dimensions varied across science domains, echoing previous findings on the heterogeneity of ideological and knowledge correlates of science skepticism (e.g., Rutjens et al., 2022; Rutjens et al., 2018). More specifically, while vaccination skepticism was predicted predominantly by hypothetical distance, social distance played a role in skepticism toward GM foods and genetic editing in humans, and temporal distance predicted evolution and climate change skepticism (also see Jones et al., 2017; Spence et al., 2012). Although further research is needed to replicate this configuration of findings and extend it to other science domains, it is worth noting that our results seem to point to a distinction between natural and earth sciences that mostly require observing natural phenomena (climate change and evolution) and biomedical sciences in which novel technologies are created (GM foods and genetic editing in humans). Regarding the first category, the perception of science as relevant predominantly for the distant future was related to higher skepticism. For the second category, perceiving scientists as dissimilar to oneself and/or inaccessible led to higher levels of skepticism. It remains an open question why this was not the case for vaccination, as this domain also falls under the umbrella of biomedical sciences and entails creating novel technologies. A possible reason for this could be the high salience of vaccination information in the media due to the COVID-19 pandemic at the time when the studies were run.

### Beyond CLT and General Science Attitudes

Psychological distance is a concept mostly studied within the framework of CLT. Although we use the dimensions of psychological distance developed within this framework, we are agnostic about whether the current results reflect one of the core mechanisms proposed by CLT—that distance relates to construal levels. Although mentally construing science on a concrete level might very well facilitate science acceptance, due to, for example, perceiving science claims as more subjectively true (Hansen & Wänke, 2010), we focus on psychological distance to science specifically. This echoes recent calls for the study of psychological distance independently of construal levels (Maglio, 2020), as a broader phenomenon that can have many other potential downstream consequences and applications (Brügger, 2020). More specifically, we posit and show that perceiving science as distant, that is, as evaluating science as a hypothetical undertaking happening in faraway places, directed toward the future and conducted by dissimilar and unapproachable people, directly relates to negative evaluations of science in specific domains.

As demonstrated by its incremental validity (Study 4) in predicting science skepticism over and beyond two general science attitude scales, PSYDISC offers a valuable novel perspective on science skepticism. While Faith in Science is focused on evaluations of the epistemic value of science, the NPSS is largely geared toward measuring negative attitudes toward science. In contrast, PSYDISC taps into perceptions of one’s *personal relation* to science, that is, how the individual positions science in relation to oneself. Moreover, we posit that, unlike epistemic evaluations of science and straightforward negative attitudes toward science, PSYDISC hints at a more attainable strategy to curb skepticism—portraying science as closer to oneself might prove easier than attempting to address highly valenced attitudes.

### PSYDISC Beyond Science Skepticism

Besides the main purpose of PSYDISC—predicting and potentially illuminating ways to reduce science skepticism (e.g., Zarzeczna et al., 2022)—we demonstrated that higher PSYDISC also predicts behavioral outcomes related to skepticism surrounding science domains (i.e., lower COVID-19 vaccination uptake). More specifically, we showed that PSYDISC, measured several months prior, predicts a lower likelihood of being fully vaccinated against COVID-19, while controlling for demographics, ideological variables, and science knowledge and understanding. In addition, we showed that vaccinated individuals’ subjective hesitancy in making the decision to receive a COVID-19 vaccine was positively related to PSYDISC. These results underline the importance of public perceptions of science for highly consequential behaviors.

Moreover, PSYDISC could also prove useful for other lines of research. First, PSYDISC could be used to better understand and explain a set of beliefs related to, but conceptually distinct from science skepticism—pseudoscientific beliefs. Given that PSYDISC predicts *rejection* of scientific findings, it would be important to test whether it also predicts *acceptance* of scientifically unsupported beliefs (e.g., extrasensory abilities) and practices (e.g., alternative medicine). Second, to the best of our knowledge, our research is the first to highlight a novel application of psychological distance—perceptions of a social institution and cultural construct (i.e., science). Therefore, PSYDISC can serve as a guide for utilizing psychological distance to measure specific perceptions of other social institutions, such as government, rule of law, or religion, which might prove useful in predicting attitudes toward such institutions, as well as their downstream consequences.

### Limitations and Future Directions

This work has some limitations which could also serve as guidelines for future studies. Importantly, the studies are all correlational and therefore do not directly test the malleability of PSYDISC. Although we posit that the PSYDISC scale captures relatively stable general perceptions of science, which are likely formed as a consequence of various social-cultural factors (such as socioeconomic status, education, access and exposure to science information, and scientific role models), we also maintain that distance to science—particularly within specific domains—can be experimentally manipulated. Initial work supports this notion by demonstrating that framing science findings in the domains of genetic editing and nanotechnology as psychologically close reduces science skepticism in these domains (Zarzeczna et al., 2022). Further research is needed to experimentally test the generalizability of these findings to other science domains.

Second, being mindful of survey length and not having previously validated scales for some of the constructs we measured, we had to rely on one-item (e.g., religiosity) and/or newly created measures (e.g., personal relevance of science) for some of the variables. Even though we maintain our measures were face-valid and internally consistent, future research should scrutinize these results using more elaborate and/or more validated measures where possible (Flake et al., 2017).

Finally, we showed that the PSYDISC scale is reliable and that its structure is comparable across two countries, which shows promise that the scale is useful beyond the context of one specific country. However, future studies should test the scale in a broad range of countries (including non-WEIRD countries; see Apicella et al., 2020) to assess its broader generalizability.

## Conclusion

Given the detrimental societal consequences of science skepticism, it is imperative to advance understanding of its psychological antecedents. In this paper, we present evidence for the reliability, construct, and predictive validity of a novel scale that measures psychological distance to science: PSYDISC. Most importantly, the results point to the value of PSYDISC as a unifying framework for understanding science skepticism and a robust predictor of science skepticism across domains.

## Supplemental Material

sj-docx-1-psp-10.1177_01461672221118184 – Supplemental material for Psychological Distance to Science as a Predictor of Science Skepticism Across Domains Click here for additional data file. Supplemental material, sj-docx-1-psp-10.1177_01461672221118184 for Psychological Distance to Science as a Predictor of Science Skepticism Across Domains by Bojana Većkalov, Natalia Zarzeczna, Jonathon McPhetres, Frenk van Harreveld and Bastiaan T. Rutjens in Personality and Social Psychology Bulletin

## Appendix

### PSYDISC Scale

Responses are given on a scale of 1 (*strongly disagree*) to 7 (*strongly agree*). Items marked with R should be reverse-coded.

#### Temporal distance

1. Most of today’s science is concerned with solving problems of the distant future.
2. Science is mainly focused on the distant future.
3. Scientists spend most of their time working on issues of the distant future.
4. We will see the impact of science more in the distant future than we do in the present.

#### Social distance

1. The prospect of working as a scientist seems beyond my reach.
2. I rarely interact with scientists in real life.
3. Scientists are very different from me.
4. It would be difficult for me to meet with a scientist.

#### Hypothetical distance

1. Scientific knowledge is a reliable way to solve important issues. R
2. Science provides accurate information about the world we live in. R
3. We can rely on science to deliver results that can be implemented in real life. R
4. I can see the effects of science, whether positive or negative, on the world. R

#### Spatial distance

1. Science and scientific research play a big role in my local area. R
2. Scientific research really contributes to my local area. R
3. People from my local area don’t become scientists.
4. Very few scientists live or work in my town.

## Notes

1 We also measured several other constructs in this pilot study. However, these did not influence any analyses, results, and decisions concerning the tested PSYDISC scale. The dataset on OSF contains all measured variables.

2 We also inspected the three-factor solution, but it was suboptimal: the third factor had high loadings (>.40) from items tapping into spatial, temporal, and hypothetical distance.

3 We compared this with the factor solution after an Oblimin (delta = 0) instead of a Promax rotation. Aside from factor order, we found no meaningful differences in factor content. We therefore proceeded with the Promax pattern matrix for item selection.

4 More items loaded well on these two factors, but we opted for four keeping in mind resource constraints in psychological survey research.

5 RMSEA-based power analysis for the predicted CFA model based on 90% confidence intervals, as recommended in Jak et al. (2020), indicated a minimum of 259 participants.

6 The sample, materials, as well as analyses were preregistered. However, we slightly deviated from the preregistered CFA model—in addition to four first-order factors, we tested a model which also includes a higher-order distance factor. There were no other deviations from the preregistration.

7 We did not include CoS in the analyses due to the substantial above-mentioned overlap with NPSS, which was also reflected in high (>.2) VIFs in the regressions with CoS included. We chose NPSS over CoS due to NPSS reflecting a broader range of negative science attitudes.

8 We could not conduct our preregistered analyses for vaccination status of children because we did not have a sufficient number of parents with children eligible for vaccination. All data and materials are reported on OSF.
