# Supplementary material for: Psychological Distance to Science as a Predictor of Science Skepticism Across Domains
Source: Pers Soc Psychol Bull. 2022 Sep 3;50(1):18–37. doi: 10.1177/01461672221118184 (PMC10676051; doi:10.1177/01461672221118184)
Supplement: sj-docx-1-psp-10.1177_01461672221118184 – Supplemental material for Psychological Distance to Science as a Predictor of Science Skepticism Across Domains [file sj-docx-1-psp-10.1177_01461672221118184.docx]

**Supplemental Materials A.**

**Pilot scale development and results**

The four dimensions (i.e., temporal, spatial, social and hypothetical) of psychological distance have previously been operationalized in various ways (see Liberman & Trope, 2014). More specifically, temporal distance has been operationalized as referring either to the past or the future (vs the present moment). Spatial distance has been conceptualized as referring to faraway (vs nearby places). Social distance has been operationalized most variedly – as differentiating between others (vs. the self), dissimilarity (vs. similarity), unfamiliarity (vs. familiarity), and out-group (vs. ingroup) membership of others. Finally, hypothetical distance can refer to both uncertainty (vs. certainty), or the hypothetical (i.e., abstract; vs real) quality of the attitude object.

In creating pilot items for the PSYDISC scale, we strived to include items referring to as many operationalizations of distance as applicable in the context of perceptions of a societal institution. First, we included temporal items that refer to the past (e.g., “Compared to its past achievements, science has become less relevant”), the future (e.g., “It takes too long for a scientific discovery to be applied in practice”; implying higher relevance of science for the future), and the present (“I can’t see the effects of science on the world right now”).

Second, spatial distance items were focused on science activity in (e.g., “Very few scientists live or work in my town”) and contributions to (e.g., “Scientific research really contributes to my local area”) the local area, as well as faraway areas (e.g., “Science is generally more present in daily life in other countries than in my own").

Third, we focused the social distance items on scientists, as a social group central to science. Since this scale is intended for the general public (i.e., non-scientists), the in- (vs. outgroup) operationalization of social distance was not applicable. We instead focused on the perceived (un)familiarity (e.g., “I can easily think of examples of prominent scientists”) and (dis)similarity (e.g., “Scientists are very different from me”) of scientists. In addition, we strived to tap into the “other vs self” aspect of social distance by tapping into whether one can identify with being a scientist (e.g., “People like me rarely become scientists”).

Finally, hypothetical distance to science items focused on both the perceived *uncertainty* in science (e.g., “Scientists are always changing their minds about how the world works”; “Scientific findings often contradict one another, creating confusion”), as well as the *hypotheticality* of science, that is, science not being readily applicable to practical, real-world matters (e.g., “Science is mostly hypothetical”; “Scientific knowledge is a reliable way to solve important issues”).

**Table S1**

*Rotated factor loadings and communalities for PSYDISC items, Pilot Study.*

| Intended dimension | Item | Pattern loadings | | | |  |
| --- | --- | --- | --- | --- | --- | --- |
|  |  | **1** | 2 | **3** | **4** | *h^2^* |
| Social | **The prospect of working as a scientist seems beyond my reach.** | .91 |  | -.17 | -.14 | .63 |
| Social | **Scientists are very different from me.** | .83 |  |  |  | .60 |
| Social | People like me rarely become scientists.^1^ | .68 |  |  | .18 | .57 |
| Social | **I rarely interact with scientists in real life.** | .64 |  | -.14 | .22 | .48 |
| Social | **It would be difficult for me to meet with a scientist.** | .59 |  |  | .21 | .50 |
| Social | The prospect of working as a scientist is appealing to me. | -.55 |  | -.30 | .28 | .33 |
| Social | I doubt I could have a meaningful dialogue with a scientist. | .49 |  | .15 |  | .36 |
| Hypothetical | I am not really sure what science is or what scientists do. | .38 |  | .26 |  | .36 |
| Temporal | It takes too long for science to solve important issues. |  | .75 |  |  | .43 |
| Temporal | It takes too long for a scientific discovery to be applied in practice. |  | .73 | -.25 |  | .39 |
| Hypothetical | Scientists are always changing their minds about how the world works. |  | .61 | -.20 |  | .23 |
| Hypothetical | Science is mostly concerned with speculation that is not useful in real life. |  | .60 | .25 |  | .54 |
| Hypothetical | Scientific findings often contradict one another, creating confusion. |  | .60 |  |  | .30 |
| Hypothetical | Scientists don’t really know what they are talking about. |  | .54 | .31 | -.16 | .52 |
| Temporal | Science is mainly focused on issues that are not relevant right now. |  | .46 | .16 | .11 | .34 |
| Hypothetical | Science is mostly hypothetical. |  | .42 |  | .16 | .27 |
| Temporal | Compared to its past achievements, science has become less relevant. |  | .41 | .26 |  | .40 |
| Social | Scientists don’t value the opinions of people like me. | .33 | .38 |  |  | .40 |
| Spatial | Scientific advances mostly benefit distant places. |  | .36 | .14 | .28 | .37 |
| Social | Scientists don’t care about sharing their work with people like me. | .27 | .28 | .20 | .11 | .45 |
| Temporal | I can’t see the effects of science on the world right now. |  | .20 | .15 |  | .10 |
| Spatial | Science is generally more present in daily life in other countries than in my own. | .10 | .20 | .17 | .15 | .24 |
| Hypothetical | **Scientific knowledge is a reliable way to solve important issues.** |  |  | -.82 |  | .57 |
| Hypothetical | **Science provides accurate information about the world we live in.** |  |  | -.66 |  | .41 |
| Temporal | **We are seeing the effects of science, whether positive or negative, right now.** | .12 | .12 | -.64 | -.14 | .34 |
| Temporal | Everyday, I use things that are the result of science.^2^ |  | .10 | -.59 |  | .35 |
| Social | I can easily think of examples of prominent scientists. | -.43 | .25 | -.50 |  | .44 |
| Hypothetical | You can’t count on science for solving important issues. |  | .24 | .37 |  | .29 |
| Hypothetical | Science is too complicated to be useful in real life. | .21 | .35 | .35 |  | .51 |
| Spatial | **Very few scientists live or work in my town.** | .12 |  |  | .70 | .50 |
| Spatial | **Science and scientific research plays a big role in my local area.** | .13 |  | -.22 | -.66 | .42 |
| Spatial | **People from my local area don’t become scientists.** |  | .14 | -.13 | .62 | .49 |
| Spatial | **Scientific research really contributes to my local area.** |  |  | -.21 | -.53 | .38 |
| Spatial | Scientific research is conducted in institutions far away from where I live. | .17 | .12 |  | .53 | .49 |

*Note.* The extraction method was principal axis factoring with an oblique (Promax with Kaiser Normalization) rotation.

Factor loadings below .10 are omitted.

Factors in bolded numbers retained (1 and 4) or refined (3) for next study.

^1^Omitted due to high conceptual similarity with first item in the factor.

^2^ Omitted due to conceptual mismatch with other items in the factor.

**References:**

Liberman, N., & Trope, Y. (2014). Traversing psychological distance. *Trends in Cognitive Sciences*, *18*(7), 364–369. <https://doi.org/10.1016/j.tics.2014.03.001>

**Supplemental Materials B.**

**Genetic editing skepticism scale**

Please indicate the extent to which you agree or disagree with each statement using the scale below. (Response scale from 1 = *strongly disagree* to 7 = *strongly agree*)

1. The potential risks of using gene editing on humans outweigh the benefits.
2. Gene editing in humans is a safe and reliable technology.
3. I trust that only safe and reliable gene modification procedures will be made available for human use.
4. Gene editing is an important contribution to the medical treatment of genetic diseases.
5. Because there are so many unknowns, it is dangerous to manipulate the natural genetic material of humans.
6. Gene editing is ethically wrong because it interferes with natural processes.
7. Gene editing puts too much power in scientists' hands.
8. Gene editing violates important moral principles.

**Supplemental Materials C.**

**PSYDISC correlations with ideological variables, Studies 1 and 2**

**Table S2**

*PSYDISC correlations with ideological variables, Studies 1 & 2.*

|  |  | Religiosity | Religious orthodoxy | Spirituality | Conspiracy beliefs | Political  conservatism |
| --- | --- | --- | --- | --- | --- | --- |
| Study 1 | PSYDISC | .066  [-.041, .176] | .155^**^  [.045, .256] | .158^**^  [.038, .274] | .355^**^  [.230, .462] | .200^**^  [.094, .311] |
|  | Social | .029  [-.088, .134] | .113  [-.011, .214] | .154^**^  [.041, .268] | .228^**^  [.105, .341] | .143^*^  [.036, .246] |
|  | Spatial | -.014  [-.133, .105] | -.003  [-.117, .113] | .025  [-.090, .136] | .193^***^  [-.077, .306] | .129^*^  [-.008, .247] |
|  | Temporal | .092  [-.025, .215] | .180^**^  [.066, .289] | .091  [-.025, .208] | .332^***^  [.230, .423] | .156^**^  [-.033, .270] |
|  | Hypothe-tical | .106  [-.002, .216] | .169^**^  [.060, .284] | .217^***^  [.104, .328] | .268^***^  [.159, .368] | .148^*^  [.022, .266] |
|  | *M* (*SD*) | 2.07 (1.50) | 2.44 (1.51) | 2.77 (1.57) | 4.98 (2.21) | 4.31 (1.75) |
| Study 2 | PSYDISC | .146^*^  [.034, .247] | .247^**^  [.143, .352] | .124^*^  [.016, .234] | .288^**^  [.181, .388] | .257^**^  [.152, .365] |
|  | Social | .089  [-.021, .192] | .148^**^  [.038, .253] | .068  [-.003, .164] | .176^**^  [.067, .279] | .164^**^  [.044, .280] |
|  | Spatial | -.028  [-.138, .086] | .052  [-.066, .167] | -.012  [-.122, .108] | .180^**^  [.066, .296] | .156^**^  [.034, .277] |
|  | Temporal | .173^**^  [.050, .289] | .280^***^  [.161, .393] | .129^*^  [-.001, .241] | .275^***^  [.168, .378] | .204^***^  [.090, .315] |
|  | Hypothe-tical | .227^***^  [.106, .348] | .266^***^  [.162, .366] | .225^***^  [.109, .342] | .208^***^  [.107, .311] | .251^***^  [.147, .344] |
|  | *M* (*SD*) | 2.30 (1.71) | 2.72 (1.69) | 2.85 (1.71) | 5.04 (2.31) | 4.37 (1.76) |

*Note.* Pearson’s *r* with 95% bootstrapped BCa CIs [L, U]. Study 1 *N*s = 286; Study 2 *N*s = 311.

^*^ *p* < .05, ^**^ *p* < .01, ^***^ *p* < .001

**Supplemental Materials D.**

**PSYDISC predictive validity full regressions, Studies 1 & 2**

Notes for Tables S3 – S12:

- Part. *r* = Partial correlation coefficient with bootstrapped BCa 95% confidence intervals.
- Study 1 *N* = 285, Study 2 *N* = 307 due to listwise omission of incomplete cases.
- Gender: 0 = male, 1 = female

***Table S3***

*Predictive power of the full PSYDISC score for climate change skepticism, Studies 1 & 2.*

|  | Study 1 | | | | | |  | Study 2 | | | | | |
| --- | --- | --- | --- | --- | --- | --- | --- | --- | --- | --- | --- | --- | --- |
|  | Step 1 | | | Step 2 | | |  | Step 1 | | | Step 2 | | |
|  | *B*  (*SE*) | *p* | Part. *r*  [95% CI] | *B*  (*SE*) | *p* | Part. *r* [95% CI] |  | *B*  (*SE*) | *p* | Part. *r*  [95% CI] | *B*  (*SE*) | *p* | Part. *r*  [95% CI] |
| Gender ^a^ | -.05  (.12) | .689 | -.02  [-.15, .11] | -.03 (.12) | .815 | -.01  [-.15, .12] |  | .43  (.10) | < .001 | .23  [.11, .34] | .42  (.10) | < .001 | .24  [.12, .34] |
| Age | .01  (.00) | .183 | .08  [-.06, .22] | .01 (.00) | .071 | .11  [-.03, .25] |  | .00  (.00) | .613 | -.03  [-.16, .09] | .00  (.00) | .960 | .00  [-.13, .13] |
| Education | -.02  (.02) | .305 | -.06  [-.19, .07] | -.01 (.02) | .762 | -.02  [-.15, .12] |  | -.02  (.02) | .259 | -.07  [-.17, .04] | .01  (.02) | .759 | .02  [-.08, .13] |
| Religiosity | .01  (.04) | .828 | .01  [-.13, .16] | .02 (.04) | .707 | .02  [-.12, .16] |  | -.04  (.04) | .341 | -.06  [-.16, .06] | -.06  (.04) | .178 | -.08  [-.18, .04] |
| Spirituality | .06 (.04) | .140 | .09  [-.05, .23] | .05 (.04) | .235 | .07  [-.06, .21] |  | -.01  (.04) | .903 | -.01  [-.11, .10] | .00  (.04) | .998 | .00  [-.11, .11] |
| Conspiracy beliefs | .09 (.03) | .001 | .19  [.09, .29] | .07 (.03) | .008 | .16  [.06, .26] |  | .06  (.02) | .013 | .14  [.03, .25] | .05  (.02) | .041 | .12  [.01, .23] |
| Political ideology | .22 (.04) | < .001 | .35  [.23, .47] | .21 (.04) | < .001 | .34  [.22, .47] |  | .32  (.03) | < .001 | .50  [.43, .58] | .29  (.03) | < .001 | .49  [.40, .57] |
| Science knowledge | -.08 (.04) | .038 | -.12  [-.24, -.02] | -.06 (.04) | .096 | -.10  [-.22, .01] |  | -.03  (.04) | .410 | -.05  [-.14, .05] | -.01  (.03) | .809 | -.01  [-.12, .10] |
| Science understanding | -.03 (.02) | .176 | -.08  [-.21, .04] | -.02 (.03) | .471 | -.04  [-.17, .09] |  | -.05  (.02) | .028 | -.13  [-.24, -.01] | -.02  (.02) | .265 | -.06  [-.20, .07] |
| PSYDISC | – | – | – | .23 (.08) | .002 | .18  [.07, .30] |  | – | – | – | .34  (.07) | < .001 | .29  [.18, .41] |
| *Adj. R^2^* | .32 | | | .34 | | |  | .37 | | | .42 | | |
| Δ*R^2^* | – | | | 2% (*p* = .002) | | |  | – | | | 9% (*p* < .001) | | |
| *M* (*SD*) | 2.44 (1.07) | | | | | |  | 2.55 (1.09) | | | | | |

**Table S4**

*Predictive power of the PSYDISC subscales for climate change skepticism, Studies 1 & 2.*

|  | Study 1 | | | | | |  | Study 2 | | | | | |
| --- | --- | --- | --- | --- | --- | --- | --- | --- | --- | --- | --- | --- | --- |
|  | Step 1 | | | Step 2 | | |  | Step 1 | | | Step 2 | | |
|  | *B* (*SE*) | *p* | Part. *r*  [95% CI] | *B* (*SE*) | *p* | Part. *r*  [95% CI] |  | *B* (*SE)* | *p* | Part. *r*  [95% CI] | *B* (*SE)* | *p* | Part. *r*  [95% CI] |
| Gender ^a^ | -.05 (.12) | .689 | -.02  [-.15, .11] | -.01  (.11) | .905 | -.01  [-.15, .13] |  | .43  (.10) | < .001 | .23  [.11, .34] | .40  (.10) | < .001 | .23  [.11, .34] |
| Age | .01  (.00) | .183 | .08  [-.06, .22] | .01  (.00) | .141 | .09  [-.06, .23] |  | .00  (.00) | .613 | -.03  [-.16, .09] | .00  (.00) | .811 | .01  [-.12, .15] |
| Education | -.02  (.02) | .305 | -.06  [-.19, .07] | -.01  (.02) | .656 | -.03  [-.16, .11] |  | -.02  (.02) | .259 | -.07  [-.17, .04] | .00  (.02) | .931 | .01  [-.10, .11] |
| Religiosity | .01  (.04) | .828 | .01  [-.13, .16] | .01  (.04) | .821 | .01  [-.13, .16] |  | -.04  (.04) | .341 | -.06  [-.16, .06] | -.07  (.04) | .104 | -.09  [-.21, .03] |
| Spirituality | .06  (.04) | .140 | .09  [-.05, .23] | .03  (.04) | .408 | .05  [-.09, .20] |  | -.01  (.04) | .903 | -.01  [-.11, .10] | -.01  (.04) | .775 | -.02  [-.12, .10] |
| Conspiracy beliefs | .09  (.03) | .001 | .19  [.09, .29] | .06  (.03) | .032 | .13  [-.03, .22] |  | .06  (.02) | .013 | .14  [.03, .25] | .05  (.02) | .052 | .11  [.00, .23] |
| Political ideology | .22  (.04) | < .001 | .35  [.23, .47] | .21  (.04) | < .001 | .35  [.22, .47] |  | .32  (.03) | < .001 | .50  [.43, .58] | .28  (.03) | < .001 | .47  [.38, .56] |
| Science knowledge | -.08  (.04) | .038 | -.12  [-.24, -.02] | -.06  (.04) | .109 | -.10  [-.21, .01] |  | -.03  (.04) | .410 | -.05  [-.14, .05] | -.01  (.03) | .727 | -.02  [-.12, .09] |
| Science understanding | -.03  (.02) | .176 | -.08  [-.21, .04] | -.02  (.02) | .399 | -.05  [-.19, .08] |  | -.05  (.02) | .028 | -.13  [-.24, -.01] | -.03  (.04) | .148 | -.08  [-.22, .04] |
| Social distance | – | – | – | .01  (.05) | .827 | .01  [-.10, .12] |  | – | – | – | .01  (.04) | .863 | .01  [-.10, .13] |
| Spatial distance | – | – | – | .00  (.05) | .978 | .00  [-.14, .13] |  | – | – | – | .07  (.05) | .199 | .07  [-.05, .21] |
| Hypothetical distance | – | – | – | .37  (.08) | < .001 | .26  [.12, .41] |  | – | – | – | .36  (.07) | < .001 | .28  [.17, .40] |
| Temporal distance | – | – | – | .07  (.05) | .136 | .09  [-.04, .23] |  | – | – | – | .10  (.04) | .029 | .13  [.01, .25] |
| *Adj. R^2^* | .32 | | | .37 | | |  | .37 | | | .45 | | |
| Δ*R^2^* | – | | | 6% (*p* < .001) | | |  | – | | | 9% (*p* < .001) | | |
| *M* (*SD*) | 2.44 (1.07) | | | | | |  | 2.55 (1.09) | | | | | |

**Table S5**

*Predictive power of the total PSYDISC score for vaccination skepticism, Studies 1 & 2.*

|  | Study 1 | | | | | |  | Study 2 | | | | | |
| --- | --- | --- | --- | --- | --- | --- | --- | --- | --- | --- | --- | --- | --- |
|  | Step 1 | | | Step 2 | | |  | Step 1 | | | Step 2 | | |
|  | *B* (*SE)* | *p* | Part. *r*  [95% CI] | *B* (*SE)* | *p* | Part. *r*  [95% CI] |  | *B* (*SE)* | *p* | Part. *r*  [95% CI] | *B* (*SE)* | *p* | Part. *r*  [95% CI] |
| Gender ^a^ | -.19  (.12) | .111 | -.10  [-.21, .03] | -.16  (.11) | .164 | -.08  [-.19, .04] |  | .01  (.10) | .925 | .01  [-.12, .12] | .00  (.10) | .972 | .00  [-.12, .12] |
| Age | .00  (.00) | .500 | -.04  [-.16, .07] | .00  (.00) | .963 | .00  [-.12, .12] |  | -.01  (.00) | .024 | -.13  [-.23, -.04] | -.01  (.00) | .059 | -.11  [-.21, -.01] |
| Education | -.01  (.02) | .748 | -.02  [-.14, .10] | .01  (.02) | .433 | .05  [-.08, .17] |  | -.02  (.02) | .340 | -.06  [-.17, .06] | .01  (.02) | .693 | .02  [-.10, .14] |
| Religiosity | -.03  (.04) | .493 | -.04  [-.19, .10] | -.02  (.04) | .639 | -.03  [-.16, .11] |  | -.01  (.04) | .748 | -.02  [-.16, .12] | -.03  (.04) | .505 | -.04  [-.17, .09] |
| Spirituality | .13  (.04) | .002 | .19  [.05, .33] | .11  (.04) | .005 | .17  [.03, .31] |  | .07  (.04) | .136 | .09  [-.04, .22] | .07  (.04) | .096 | .10  [-.03, .23] |
| Conspiracy beliefs | .18  (.03) | < .001 | .37  [.29, .46] | .15  (.03) | < .001 | .34  [.24, .43] |  | .11  (.02) | < .001 | .25  [.14, .35] | .10  (.02) | < .001 | .23  [.12, .33] |
| Political ideology | .11  (.04) | .002 | .18  [.05, .34] | .10  (.03) | .006 | .17  [.04, .33] |  | .09  (.03) | .003 | .17  [.04, .29] | .07  (.03) | .022 | .13  [-.01, .26] |
| Science knowledge | -.13  (.04) | < .001 | -.21  [-.34, -.08] | -.11  (.04) | .003 | -.18  [-.30, -.05] |  | -.05  (.04) | .122 | -.09  [-.21, .04] | -.04  (.03) | .307 | -.06  [-.19, .08] |
| Science understanding | -.01  (.03) | .637 | -.03  [-.16, .10] | .01  (.03) | .620 | .03  [-.10, .16] |  | -.05  (.02) | .036 | -.12  [-.22, -.01] | -.02  (.02) | .283 | -.06  [-.16, .05] |
| PSYDISC | – | – | – | .35  (.07) | < .001 | .28  [.17, .37] |  | – | – | – | .32  (.07) | < .001 | .27  [.16, .38] |
| *Adj. R^2^* | .35 | | | .39 | | |  | .23 | | | .28 | | |
| Δ*R^2^* | – | | | 5% (*p* < .001) | | |  | – | | | 6% (*p* < .001) | | |
| *M* (*SD*) | 2.14 (1.08) | | | | | |  | 2.08 (.97) | | | | | |

**Table S6**

*Predictive power of PSYDISC subscales for vaccination skepticism, Studies 1 & 2.*

|  | Study 1 | | | | | |  | Study 2 | | | | | |
| --- | --- | --- | --- | --- | --- | --- | --- | --- | --- | --- | --- | --- | --- |
|  | Step 1 | | | Step 2 | | |  | Step 1 | | | Step 2 | | |
|  | *B* (*SE)* | *p* | Part. *r*  [95% CI] | *B* (*SE)* | *p* | Part. *r*  [95% CI] |  | *B* (*SE)* | *p* | Part. *r*  [95% CI] | *B* (*SE)* | *p* | Part. *r*  [95% CI] |
| Gender ^a^ | -.19  (.12) | .111 | -.10  [-.21, .03] | -.13  (.11) | .210 | -.08  [-.20, .05] |  | .01  (.10) | .925 | .01  [-.12, .12] | -.04  (.10) | .657 | -.03  [-.14, .09] |
| Age | .00  (.00) | .500 | -.04  [-.16, .07] | .00  (.00) | .809 | -.01  [-.15, .12] |  | -.01  (.00) | .024 | -.13  [-.23, .04] | -.01  (.00) | .039 | -.12  [-.22, -.02] |
| Education | -.01  (.02) | .748 | -.02  [-.14, .10] | .01  (.02) | .639 | .03  [-.10, .16] |  | -.02  (.02) | .340 | -.06  [-.17, .06] | .00  (.02) | .823 | .01  [-.11, .13] |
| Religiosity | -.03  (.04) | .493 | -.04  [-.19, .10] | -.03  (.04) | .494 | -.04  [-.18, .08] |  | -.01  (.04) | .748 | -.02  [-.16, .12] | -.04  (.04) | .320 | -.06  [-.18, .06] |
| Spirituality | .13  (.04) | .002 | .19  [.05, .33] | .10  (.04) | .011 | .15  [.01, .31] |  | .07  (.04) | .136 | .09  [-.04, .22] | .05  (.04) | .195 | .08  [-.06, .23] |
| Conspiracy beliefs | .18  (.03) | < .001 | .37  [.29, .46] | .14  (.03) | < .001 | .31  [.22, .41] |  | .11  (.02) | < .001 | .25  [.14, .35] | .10  (.02) | < .001 | .24  [.13, .34] |
| Political ideology | .11  (.04) | .002 | .18  [.05, .34] | .09  (.03) | .006 | .17  [.03, .31] |  | .09  (.03) | .003 | .17  [.04, .29] | .05  (03) | .073 | .10  [-.04, .23] |
| Science knowledge | -.13  (.04) | < .001 | -.21  [-.34, -.08] | -.11  (.03) | .002 | -.19  [-.32, -.06] |  | -.05  (.04) | .122 | -.09  [-.21, .04] | -.04  (.03) | .244 | -.07  [-.20, .07] |
| Science understanding | -.01  (.03) | .637 | -.03  [-.16, .10] | .00  (.02) | .964 | .00  [-.14, .14] |  | -.05  (.02) | .036 | -.12  [-.22, -.01] | -.03  (.02) | .127 | -.09  [-.20, .02] |
| Social distance | – | – | – | -.02  (.04) | .828 | -.03  [-.15, .07] |  | – | – | – | .04  (.04) | .351 | .05  [-.05, .15] |
| Spatial distance | – | – | – | .10  (.05) | .044 | .12  [-.02, .25] |  | – | – | – | .02  (.05) | .653 | .03  [-.08, .13] |
| Hypothetical distance | – | – | – | .46  (.08) | < .001 | .34  [.21, .45] |  | – | – | – | .48  (.07) | < .001 | .38  [.24, .48] |
| Temporal distance | – | – | – | .08  (.05) | .074 | .11  [-.02, .22] |  | – | – | – | .03  (.04) | .524 | .04  [-.08, .16] |
| *Adj. R^2^* | .35 | | | .45 | | |  | .23 | | | .35 | | |
| Δ*R^2^* | – | | | 10% (*p* < .001) | | |  | – | | | 13% (*p* < .001) | | |
| *M* (*SD*) | 2.14 (1.08) | | | | | |  | 2.08 (.97) | | | | | |

**Table S7**

*Predictive power of the PSYDISC total score for evolution skepticism, Studies 1 & 2.*

|  | Study 1 | | | | | |  | Study 2 | | | | | |
| --- | --- | --- | --- | --- | --- | --- | --- | --- | --- | --- | --- | --- | --- |
|  | Step 1 | | | Step 2 | | |  | Step 1 | | | Step 2 | | |
|  | *B* (*SE)* | *p* | Part. *r*  [95% CI] | *B* (*SE)* | *p* | Part. *r*  [95% CI] |  | *B* (*SE)* | *p* | Part. *r*  [95% CI] | *B* (*SE)* | *p* | Part. *r*  [95% CI] |
| Gender ^a^ | -.04 (.11) | .728 | -.02  [-.16, .12] | -.01 (.11) | .932 | -.01  [-.13, .13] |  | .34  (.10) | < .001 | .20  [.09, .31] | .33  (.09) | < .001 | .20  [.09, .32] |
| Age | .00 (.00) | .623 | -.03  [-.18, .13] | .00 (.00) | .794 | .02  [-.14, .17] |  | .00  (.00) | .792 | .02  [-.10, .14] | .00  (.00) | .412 | .05  [-.08, .17] |
| Education | -.03 (.02) | .066 | -.11  [-.24, .01] | -.01 (.02) | .473 | -.04  [-.17, .07] |  | .00  (.02) | .803 | .01  [-.11, .13] | .03  (.02) | .059 | .11  [-.01, .22] |
| Religiosity | .14 (.04) | < .001 | .20  [.05, .37] | .15 (.04) | < .001 | .22  [.08, .39] |  | .07  (.04) | .071 | .10  [-.01, .23] | .06  (.04) | .134 | .09  [-.03 .21] |
| Spirituality | .06 (.04) | .120 | .09  [-.04, .23] | .04 (.04) | .261 | .07  [-.07, .20] |  | .09  (.04) | .023 | .13  [.03, .25] | .10  (.04) | .011 | .15  [.04, .27] |
| Conspiracy beliefs | .02 (.03) | .474 | .04  [-.08, .18] | -.01 (.02) | .816 | -.01  [-.13, .12] |  | .03  (.02) | .210 | .07  [-.06, .19] | .02  (.02) | .474 | .04  [-.09, .16] |
| Political ideology | .10 (.03) | .003 | .18  [.06, .29] | .09 (.03) | .008 | .16  [.04, .24] |  | .14  (.03) | < .001 | .26  [.14, .40] | .11  (.03) | < .001 | .23  [.10, .37] |
| Science knowledge^[[1]](#footnote-1)^ | -.19 (.04) | < .001 | -.30  [-.41, -.20] | -.16 (.03) | < .001 | -.28  [-.39, -.16] |  | -.12  (.03) | < .001 | -.21  [-.31, -.10] | -.10  (.03) | .001 | -.18  [-.29, -.07] |
| Science understanding | -.05 (.02) | .024 | -.14  [-.25, -.02] | -.03 (.02) | .196 | -.08  [-.19, .04] |  | -.05  (.02) | .013 | -.14  [-.26, -.03] | -.03  (.02) | .204 | -.07  [-.19, .03] |
| PSYDISC | – | – | – | .35 (.07) | < .001 | .29  [.18, .38] |  | – | – | – | .36  (.06) | < .001 | .32  [.21, .43] |
| *Adj. R^2^* | .33 | | | .38 | | |  | .37 | | | .44 | | |
| Δ*R^2^* | – | | | 5% (*p* < .001) | | |  | – | | | 6% (*p* < .001) | | |
| *M* (*SD*) | 2.10 (1.02) | | | | | |  | 2.14 (1.02) | | | | | |

**Table S8**

*Predictive power of the PSYDISC subscales for evolution skepticism, Studies 1 & 2.*

|  | Study 1 | | | | | |  | Study 2 | | | | | |
| --- | --- | --- | --- | --- | --- | --- | --- | --- | --- | --- | --- | --- | --- |
|  | Step 1 | | | Step 2 | | |  | Step 1 | | | Step 2 | | |
|  | *B* (*SE)* | *p* | Part. *r*  [95% CI] | *B* (*SE)* | *p* | Part. *r*  [95% CI] |  | *B* (*SE)* | *p* | Part. *r*  [95% CI] | *B* (*SE)* | *p* | Part. *r* [95% CI] |
| Gender ^a^ | -.04 (.11) | .728 | -.02  [-.16, .12] | .01 (.10) | .945 | .00  [-.13, .14] |  | .34  (.10) | < .001 | .20  [.09, .31] | .30  (.09) | .001 | .19  [.09, .31] |
| Age | .00 (.00) | .623 | -.03  [-.18, .13] | .00 (.00) | .942 | .00  [-.14, .14] |  | .00  (.00) | .792 | .02  [-.10, .14] | .00  (.00) | .363 | .05  [-.07, .17] |
| Education | -.03 (.02) | .066 | -.11  [-.24, .01] | -.02 (.02) | .343 | -.06  [-.18, .08] |  | .00  (.02) | .803 | .01  [-.11, .13] | .03  (.02) | .081 | .10  [-.02, .22] |
| Religiosity | .14 (.04) | < .001 | .20  [.05, .37] | .14 (.04) | < .001 | .22  [.06, .39] |  | .07  (.04) | .071 | .10  [-.01, .23] | .04  (.04) | .279 | .06  [-.06, .18] |
| Spirituality | .06 (.04) | .120 | .09  [-.04, .23] | .03 (.04) | .444 | .05  [-.10, .19] |  | .09  (.04) | .023 | .13  [.03, .25] | .09  (.04) | .019 | .14  [.03, .26] |
| Conspiracy beliefs | .02 (.03) | .474 | .04  [-.08, .18] | -.02 (.02) | .407 | -.05  [-.17, .09] |  | .03  (.02) | .210 | .07  [-.06, .19] | .02  (.02) | .484 | .04  [-.10, .28] |
| Political ideology | .10 (.03) | .003 | .18  [.06, .29] | .09 (.03) | .007 | .16  [.04, .28] |  | .14  (.03) | < .001 | .26  [.14, .40] | .10  (.03) | < .001 | .21  [.09, .35] |
| Science knowledge^[[2]](#footnote-2)^ | -.19 (.04) | < .001 | -.30  [-.41, -.20] | -.16 (.03) | < .001 | -.28  [-.39, -.16] |  | -.12  (.03) | < .001 | -.21  [-.31, -.10] | -.10  (.03) | .002 | -.18  [-.29, -.06] |
| Science understanding | -.05 (.02) | .024 | -.14  [-.25, -.02] | -.04 (.02) | .118 | -.09  [-.21, .02] |  | -.05  (.02) | .013 | -.14  [-.26, -.03] | -.03  (.02) | .137 | -.09  [-.19, .02] |
| Social distance | – | – | – | .02 (.04) | .640 | .03  [-.09, .13] |  | – | – | – | .06  (.04) | .071 | .11  [-.02, .23] |
| Spatial distance | – | – | – | .06 (.05) | .261 | .07  [-.06, .20] |  | – | – | – | .00  (.05) | .924 | -.01  [-.13, .11] |
| Hypothetical distance | – | – | – | .42 (.08) | < .001 | .32  [.18, .45] |  | – | – | – | .36  (.07) | < .001 | .31  [.19, .42] |
| Temporal distance | – | – | – | .09 (.04) | .040 | .12  [.01, .23] |  | – | – | – | .11  (.04) | .009 | .15  [.02, .29] |
| *Adj. R^2^* | .33 | | | .42 | | |  | .37 | | | .47 | | |
| Δ*R^2^* | – | | | 10% (*p* < .001) | | |  | – | | | 10% (*p* < .001) | | |
| *M* (*SD*) | 2.10 (1.02) | | | | | |  | 2.14 (1.02) | | | | | |

**Table S9**

*Predictive power of the total PSYDISC score for genetically modified foods, Studies 1 & 2.*

|  | Study 1 | | | | | |  | Study 2 | | | | | |
| --- | --- | --- | --- | --- | --- | --- | --- | --- | --- | --- | --- | --- | --- |
|  | Step 1 | | | Step 2 | | |  | Step 1 | | | Step 2 | | |
|  | *B* (*SE)* | *p* | Part. *r*  [95% CI] | *B*  (*SE)* | *p* | Part. *r*  [95% CI] |  | *B*  (*SE)* | *p* | Part. *r*  [95% CI] | *B*  (*SE)* | *p* | Part. *r*  [95% CI] |
| Gender ^a^ | .12 (.14) | .401 | .05  [-.07, .18] | .16  (.13) | .226 | .07  [-.05, .19] |  | .24  (.13) | .069 | .11  [-.02, .23] | .24  (.13) | .071 | .10  [-.02, .23] |
| Age | .02 (.01) | < .001 | .22  [.11, .33] | .02  (.00) | <.001 | .28  [.18, .39] |  | .01  (.01) | .089 | .10  [-.02, .21] | .01  (.00) | .053 | .11  [.00, .23] |
| Education | .03 (.02) | .189 | .08  [-.05, .19] | .06  (.05) | .007 | .16  [.04, .28] |  | .00  (.00) | .834 | .01  [-.09, .11] | .02  (.02) | .370 | .05  [-.05, .16] |
| Religiosity | .00  (05) | .979 | .00  [-.12, .12] | .02  (.05) | .748 | .02  [-.10, .14] |  | -.11  (.06) | .045 | -.12  [-.21, - .02] | -.12  (.05) | .029 | -.13  [-.22, -.03] |
| Spirituality | .23  (.05) | < .001 | .27  [.15, .39] | .21  (.05) | <.001 | .26  [.13, .38] |  | .22  (.06) | <.001 | .22  [.10, .32] | .22  (.06) | <.001 | .22  [.11, .33] |
| Conspiracy beliefs | .14  (.03) | < .001 | .25  [.13, .38] | .10  (.03) | <.001 | .20  [.07, .33] |  | .10  (.03) | .002 | .17  [.06, .28] | .09  (.03) | .005 | .16  [.05, .27] |
| Political ideology | .03  (.04) | .508 | .04  [-.09, .17] | .01  (.04) | .855 | .01  [-.13, .15] |  | .07  (.04) | .093 | .10  [-.03, .22] | .05  (.04) | .191 | .08  [-.05, .20] |
| Science knowledge | -.05  (.04) | .262 | -.07  [-.19, .04] | -.02  (.04) | .716 | -.02  [-.14, .08] |  | -.09  (.05) | .059 | -.11  [-.21, .00] | -.07  (.05) | .109 | -.09  [-.19, .01] |
| Science understanding | -.07  (.03) | .024 | -.14  [-.25, -.01] | -.03  (.03) | .247 | -.07  [-.19, .04] |  | -.04  (.03) | .150 | -.08  [-.21, .04] | -.03  (.03) | .382 | -.05  [-.18, .07] |
| PSYDISC | – | – | – | .50  (.09) | <.001 | .33  [.22, .43] |  | – | – | – | .22  (.09) | .013 | .14  [.02, .26] |
| *Adj. R^2^* | .29 | | | .38 | | |  | .21 | | | .22 | | |
| Δ*R^2^* | – | | | 10% (*p* < .001) | | |  | 2% (*p* = .013) | | | | | |
| *M* (*SD*) | 3.91 (1.24) | | | | | |  | 3.68 (1.23) | | | | | |

**Table S10**

*Predictive power of PSYDISC subscales for genetically modified foods, Studies 1 & 2.*

|  | Study 1 | | | | | |  | Study 2 | | | | | |
| --- | --- | --- | --- | --- | --- | --- | --- | --- | --- | --- | --- | --- | --- |
|  | Step 1 | | | Step 2 | | |  | Step 1 | | | Step 2 | | |
|  | *B* (*SE)* | *p* | Part. *r*  [95% CI] | *B* (*SE)* | *p* | Part. *r*  [95% CI] |  | *B* (*SE)* | *p* | Part. *r*  [95% CI] | *B* (*SE)* | *p* | Part. *r*  [95% CI] |
| Gender ^a^ | .12 (.14) | .401 | .05  [-.07, .18] | .18  (.13) | .159 | .09  [-.04, .21] |  | .24  (.13) | .069 | .11  [-.02, .23] | .20  (.13) | .136 | .09  [-.04, .22] |
| Age | .02 (.01) | < .001 | .22  [.11, .33] | .02  (.00) | < .001 | .24  [-.13, .35] |  | .01  (.01) | .089 | .10  [-.02, .21] | .01  (.01) | .121 | .09  [-.03, .21] |
| Education | .03 (.02) | .189 | .08  [-.05, .19] | .06  (.02) | .007 | .16  [.04, .27] |  | .00  (.00) | .834 | .01  [-.09, .11] | .02  (.02) | .376 | .05  [-.05, .15] |
| Religiosity | .00  . (05) | .979 | .00  [-.12, .12] | .02  (.05) | .747 | .02  [-.10, .15] |  | -.11  (.06) | .045 | -.12  [-.21, -.02] | -.13  (.05) | .015 | -.14  [-.24, -.04] |
| Spirituality | .23  (.05) | < .001 | .27  [.15, .39] | .18  (.05) | < .001 | .23  [.09, .36] |  | .22  (.06) | <.001 | .22  [.10, .32] | .21  (.06) | <.001 | .22  [.10, .32] |
| Conspiracy beliefs | .14  (.03) | < .001 | .25  [.13, .38] | .10  (.03) | .002 | .19  [.07, .31] |  | .10  (.03) | .002 | .17  [.06, .28] | .09  (.03) | .004 | .17  [.06, .24] |
| Political ideology | .03  (.04) | .508 | .04  [-.09, .17] | .02 (.04) | .705 | .02  [-.12, .16] |  | .07  (.04) | .093 | .10  [-.03, .22] | .05  (.04) | .223 | .07  [-.06, .21] |
| Science knowledge | -.05  (.04) | .262 | -.07  [-.19, .04] | -.02  (.04) | .692 | -.02  [-.13, .08] |  | -.09  (.05) | .059 | -.11  [-.21, .00] | -.06  (.05) | .172 | -.08  [-.18, .03] |
| Science understanding | -.07  (.03) | .024 | -.14  [-.25, -.01] | -.04  (.03) | .178 | -.08  [-.20, .03] |  | -.04  (.03) | .150 | -.08  [-.21, .04] | -.03  (.03) | .360 | -.05  [-.18, .07] |
| Social distance | – | – | – | .12  (.05) | .017 | .14  [-.02, .26] |  | – | – | – | .11  (.05) | .036 | .12  [.00, .27] |
| Spatial distance | – | – | – | .10 (.06) | .105 | .10  [-.03, .24] |  | – | – | – | -.08  (.07) | .227 | -.07  [-.18, .06] |
| Hypothetical distance | – | – | – | .41  (.09) | < .001 | .26  [.15, .36] |  | – | – | – | .29  (.09) | .002 | .18  [.06, .27] |
| Temporal distance | – | – | – | .04  (.05) | .422 | .05  [-.07, .16] |  | – | – | – | .02  (.06) | .766 | .02  [-.11, .15] |
| *Adj. R^2^* | .29 | | | .36 | | |  | .21 | | | .24 | | |
| Δ*R^2^* | – | | | 8% (*p* < .001) | | |  | 4% (*p* = .005) | | | | | |
| *M* (*SD*) | 3.91 (1.24) | | | | | |  | 3.68 (1.23) | | | | | |

**Table S11**

*Predictive power of the total PSYDISC score for skepticism to genetic editing in humans, Study 2.*

|  | Step 1 | | |  | Step 2 | | |
| --- | --- | --- | --- | --- | --- | --- | --- |
|  | *B* (*SE)* | *p* | Part. *r*  [95% CI] |  | *B* (*SE)* | *p* | Part. *r*  [95% CI] |
| Gender ^a^ | .26 (.11) | .021 | .13 [.02, .25] |  | .25 (.11) | .020 | .13 [.02, .25] |
| Age | -.01 (.00) | .146 | -.08 [-.19, .02] |  | .00 (.00) | .297 | -.06 [-.17, .04] |
| Education | -.01 (.02) | .583 | -.03 [-.14, .08] |  | .02 (.02) | .392 | .05 [-.05, .16] |
| Religiosity | -.02 (.05) | .739 | -.02 [-.13, .09] |  | -.03 (.04) | .488 | -.04 [-.15, .07] |
| Spirituality | .11 (.05) | .021 | .13 [.02, .25] |  | .12 (.05) | .012 | .15 [.03, .26] |
| Conspiracy beliefs | .06 (.03) | .015 | .14 [.02, .26] |  | .05 (.03) | .046 | .12 [.00, .25] |
| Political ideology | .09 (.03) | .007 | .16 [.01, .29] |  | .07 (.03) | .042 | .12 [-.02, .25] |
| Science knowledge | -.03 (.04) | .423 | -.05 [-.15, .07] |  | -.01 (.04) | .817 | -.01 [-.13, .11] |
| Science understanding | -.05 (.02) | .040 | -.12 [-.22, -.00] |  | -.02 (.02) | .325 | -.06 [-.17, .06] |
| PSYDISC | – | – | – |  | .36 (.07) | < .001 | .28 [.13, .41] |
| *Adj. R^2^* | .18 | | |  | .24 | | |
| Δ*R^2^* | – | | |  | 6% (*p* < .001) | | |
| *M* (*SD*) | 3.93 (1.02) | | | | | | |

**Table S12**

*Predictive power of PSYDISC subscales for skepticism to genetic editing in humans, Study 2.*

|  | Step 1 | | |  | Step 2 | | |
| --- | --- | --- | --- | --- | --- | --- | --- |
|  | *B* (*SE)* | *p* | Part. *r*  [95% CI] |  | *B* (*SE)* | *p* | Part. *r*  [95% C*I*] |
| Gender ^a^ | .26 (.11) | .021 | .13 [.02, .25] |  | .23 (.11) | .035 | .12 [.00, .24] |
| Age | -.01 (.00) | .146 | -.08 [-.19, .02] |  | -.01 (.00) | .197 | -.08 [-.18, .04] |
| Education | -.01 (.02) | .583 | -.03 [-.14, .08] |  | .02 (.02) | .396 | .05 [-.05, .16] |
| Religiosity | -.02 (.05) | .739 | -.02 [-.13, .09] |  | -.03 (.05) | .463 | -.04 [-.15, .07] |
| Spirituality | .11 (.05) | .021 | .13 [.02, .25] |  | .11 (.05) | .019 | .14 [.02, .25] |
| Conspiracy beliefs | .06 (.03) | .015 | .14 [.02, .26] |  | .05 (.03) | .042 | .12 [.00, .24] |
| Political ideology | .09 (.03) | .007 | .16 [.01, .29] |  | .06 (.03) | .059 | .11 [-.03, .24] |
| Science knowledge | -.03 (.04) | .423 | -.05 [-.15, .07] |  | -.01 (.04) | .783 | -.02 [-.13, .10] |
| Science understanding | -.05 (.02) | .040 | -.12 [-.22, -.00] |  | -.03 (.02) | .266 | -.06 [-.17, .06] |
| Social distance | – | – | – |  | .10 (.04) | .021 | .13 [.01, .27] |
| Spatial distance | – | – | – |  | .06 (.06) | .270 | .06 [-.08, .20] |
| Hypothetical distance | – | – | – |  | .25 (.08) | .002 | .18 [.07, .29] |
| Temporal distance | – | – | – |  | .03 (.05) | .480 | .04 [-.08, .18] |
| *Adj. R^2^* | .18 | | |  | .25 | | |
| Δ*R^2^* | – | | |  | 8% (*p* < .001) | | |
| *M* (*SD*) | 3.93 (1.02) | | | | | | |

**Table S13**

*Predictive power of the total PSYDISC score for skepticism across domains, without covariates, Studies 1 and 2.*

|  |  | **Climate change** | **Vaccination** | **Evolution** | **GM foods** | **Genetic editing** |
| --- | --- | --- | --- | --- | --- | --- |
| **Study 1** | *B* (*SE*) | .47 (.07) | .61 (.07) | .56 (.07) | .61 (.08) | / |
|  | *p* | < .001 | < .001 | < .001 | < .001 |  |
|  | *r* [95% CI] | .35  [.25, .44] | .46  [.36, .54] | .44  [.34, .54] | .40  [.29, .49] |  |
| **Study 2** | *B* (*SE*) | .57 (.07) | .48 (.06) | .54 (.06) | .34 (.08) | .47 (.06) |
|  | *p* | <.001 | < .001 | < .001 | <.001 | < .001 |
|  | *r* [95% CI] | .44  [.33, .54] | .42  [.33, .51] | .45  [.36, .54] | .25  [.13, .36] | .39  [.28, .48] |

Note. Study 1 *N* = 286, Study 2 *N* = 311 due to listwise omission of incomplete cases

**Table S14**

*Predictive power of the PSYDISC subscale scores for skepticism across domains, without covariates, Studies 1 and 2.*

|  |  | **Hypothetical** | | | **Social** | | | **Temporal** | | | **Spatial** | | |
| --- | --- | --- | --- | --- | --- | --- | --- | --- | --- | --- | --- | --- | --- |
|  |  | *B*  (*SE*) | *p* | *Part. r*  [*95% CI*] | *B*  (*SE)* | *p* | *Part. r*  [*95% CI*] | *B*  (*SE*) | *p* | *Part. r*  [*95% CI*] | *B*  (*SE*) | *p* | *Part. r*  [*95% CI*] |
| **Climate change** | Study 1 | .52 (.09) | < .001 | .32  [.21, .44] | .08 (.05) | .118 | .09  [-.02, .21] | .14 | .007 | .16  [.03, .28] | -.01 | .889 | -.01  [-.12, .11] |
|  | Study 2 | .51 (.08) | < .001 | .34  [.24, .43] | .06 (.04) | .186 | .08  [-.04, .19] | .15 (.05) | .001 | .19  [.07, .30] | .08 (.06) | .148 | .08  [-.04, .20] |
| **Vaccination** | Study 1 | .64  (.09) | < .001 | .41  [.29, .52] | .03 (.05) | .508 | .04  [-.07, .15] | .18 (.05) | <.001 | .23  [.11, .34] | .10 (.06) | .087 | .10  [.24, .47] |
|  | Study 2 | .54 (.07) | < .001 | .40  [.28, .52] | .05 (.04) | .177 | .08  [-.03, .18] | .11 | < .001 | .16  [.05, .26] | .04 (.05) | .487 | .04  [-.07, .16] |
| **Evolution** | Study 1 | .53 (.08) | <.001 | .36  [.24, .47] | .09 (.04) | .039 | .12  [.02, .23] | .17 (.05) | <.001 | .22  [.11, .33] | .02 (.06) | .749 | .02  [-.11, .16] |
|  | Study 2 | .53 (.07) | <.001 | .39  [.29, .49] | .12 (.04) | .001 | .18  [.06, .31] | .18 (.04) | <.001 | .23  [.11, .35] | -.07 | .209 | -.08  [-.19, .04] |
| **GM foods** | Study 1 | .61 (.10) | <.001 | .34  [.23, .43] | .18 (.05) | <.001 | .20 [.08, .32] | .06 (.06) | .314 | .06  [-.05, .18] | .03 (.07) | .625 | .03 [-.10, .15] |
|  | Study 2 | .42 (.10) | <.001 | .24  [.13, .34] | .17 (.05) | .001 | .18  [.06, .29] | .05 (.06) | .409 | .05  [-.08, .17] | -.12 (.07) | .085 | -.10  [-.23, .03] |
| **Genetic editing** | Study 2 | .34 (.08) | <.001 | .24  [.13, .35] | .12 (.04) | .005 | .16  [.03, .27] | .09 (.05) | .048 | .11  [.00, .22] | .05 (.06) | .423 | .05  [-.08, .17] |

Note. Study 1 *N* = 286, Study 2 *N* = 311 due to listwise omission of incomplete cases

*Part. r* = partial correlation coefficient with bootstrapped BCa 95% confidence intervals.

**Supplemental Materials E.**

**Study 3: US confirmatory factor analysis**

**Measurement invariance testing**

We conducted measurement invariance testing in order to examine whether PSYDISC measures an equivalent construct across countries (i.e., UK and US). Measurement invariance testing is done in several steps, with increasingly stricter criteria for equivalence (see Putnick & Bornstein, 2016). We tested and report here the first three levels of invariance testing, sufficient for comparing scores across groups: configural (equivalence of model form); metric (equivalence of factor loadings) and scalar (equivalence of item intercepts) invariance. Following recommendations from Chen (2007), in addition to the non-significance of the Δχ2 test, we also estimated changes in CFI, RMSEA and SRMR fit indices. More specifically, a change in CFI of ≥ .01, in addition to a change in the RMSEA of ≥ .015 or a change in SRMR of ≥ .03 (for metric invariance) and ≥ .01 (for scalar invariance) would indicate a significant deterioration in model fit. As can be seen in Table S13, our scale met all criteria for full scalar invariance, indicating group comparisons in PSYDISC scores can be made between the US and the UK.

**Table S15**

*Measurement invariance across UK (Study 2) and US (Study 3) samples*

| Level of invariance | χ^2^  (*df*) | Δχ^2^  (Δ*df*) | CFI | ΔCFI | RMSEA | ΔRMSEA | SRMR | ΔSRMR |
| --- | --- | --- | --- | --- | --- | --- | --- | --- |
| Configural | 336.86^***^  (198) |  | .961 |  | 0.052 |  | 0.06 |  |
| Metric | 344.25^***^  (213) | 6.33  (15) | .963 | .002 | 0.049 | -.003 | 0.066 | .002 |
| Scalar | 361.65^***^ (224) | 17.38  (11) | .962 | -.002 | 0.049 | 0 | 0.067 | .002 |

*Note. N = 582; N_UK_ = 311; N_Us_ = 271.*

*^***^ p < .001*

**References:**

Chen, F. F. (2007). Sensitivity of Goodness of Fit Indexes to Lack of Measurement Invariance. *Structural Equation Modeling: A Multidisciplinary Journal*, 14(*3*), 464–504. <https://doi.org/10.1080/10705510701301834>

Putnick, D. L., & Bornstein, M. H. (2016). Measurement invariance conventions and reporting: The state of the art and future directions for psychological research. *Developmental Review*, 41, 71–90. <https://doi.org/10.1016/j.dr.2016.06.004>

**Table S16**

*Study 3: PSYDISC intercorrelations and correlations with personal relevance of science, distance slider, religiosity and political ideology.*

|  | Social | Spatial | Temporal | Hypothetical | Personal relevance of science | Distance slider | Religiosity | Political ideology |
| --- | --- | --- | --- | --- | --- | --- | --- | --- |
| PSYDISC | .784^***^  [.736, .827] | .697^***^  [.634, .753] | .680^***^  [.616, .737] | .508^***^  [.426, .585] | -.634^***^  [-.699, -.571] | .522^***^  [.429, .603] | .185^**^  [.060, .305] | .229^***^  [.114, .338] |
| Social | – | .406^***^  [.283, .517] | .337^***^  [.217, .453] | .247^***^  [.150, .349] | -.543^***^  [-.629, -.452] | .496^***^  [.367, .602] | .091  [-.034, .213] | .075  [-.054, .201] |
| Spatial |  | – | .199^***^  [.084, .317] | .306^***^  [.200, .408] | -.339^***^  [-.443, -.236] | .333^***^  [.220, .444] | .083  [-.035, .202] | .107  [-.015, .233] |
| Temporal |  |  | – | .158^***^  [.043, .272] | -.390^***^  [-.479, .286] | .275^***^  [.160, .382] | .187^**^  [.065, .300] | .200^***^  [.080, .318] |
| Hypothetical |  |  |  | – | -.483^***^  [-.581, .391] | .298^***^  [.199, .400] | .160^**^  [.057, .268] | .326^***^  [.216, .427] |
| *M (SD)* | 3.61 (1.43) | 3.10 (1.20) | 3.66 (1.39) | 1.84 (.72) | 6.03 (.90) | 31.85 (24.20) | 2.75 (1.90) | 3.71 (2.23) |

*Note.* Pearson’s *r* with 95% bootstrapped BCa CIs [L, U]. *N*s = 271.

^*^ *p* < .05, ^**^ *p* < .01, ^***^ *p* < .001.

**Supplemental Materials F.**

**PSYDISC prospective predictive validity for COVID-19 vaccination outcomes, Study 5**

**Table S17**

*Predictive power of PSYDISC for COVID-19 vaccination status, Study 5*^[[3]](#footnote-3)^*.*

|  | Step 1 | | | | Step 2a | | |  | Step2b | | |
| --- | --- | --- | --- | --- | --- | --- | --- | --- | --- | --- | --- |
|  | *B* | *OR* [95% CI] | *p* |  | *B* | *OR* [95% CI] | *p* |  | *B* | *OR* [95% CI] | *p* |
| Gender ^a^ | .30 | 1.34 [.72, 2.51] | .355 |  | .31 | 1.36 [.72, 2.57] | .337 |  | .36 | 1.43 [.75, 2.73] | .280 |
| Age | .03 | 1.03 [.96, 1.17] | .029 |  | .02 | 1.02 [.99, 1.05] | .064 |  | .03 | 1.03 [1.01, 1.06] | .020 |
| Education | .06 | 1.06 [81, 1.27] | .219 |  | .03 | 1.03 [.93, 1.14] | .591 |  | .02 | 1.02 [.92, 1.13] | .741 |
| Religiosity | .02 | 1.02 [-.18, .12] | .892 |  | .01 | 1.01 [.80, 1.27] | .936 |  | -.01 | .99 [.79, 1.25] | .943 |
| Spirituality | -.08 | .92 [.73, 1.16] | .479 |  | -.06 | .94 [.74, 1.19] | .609 |  | -.002 | .99 [.78, 1.27] | .978 |
| Conspiracy beliefs | -.29 | .75 [.63, .88] | <.001 |  | -.28 | .76 [.64, .90] | .001 |  | -.28 | .76 [.64, .90] | .001 |
| Political ideology | -.09 | .91 [.75, 1.11] | .367 |  | -.06 | .94 [.77, 1.15] | .552 |  | -.06 | .94 [.76, 1.16] | .570 |
| Science knowledge | .04 | 1.05 [.86, 1.27] | .656 |  | .01 | 1.01 [.83, 1.22] | .927 |  | .05 | 1.05 [.86, 1.29] | .612 |
| Science understanding | .02 | 1.02 [.90, 1.16] | .781 |  | -.01 | .99 [.86, 1.13] | .851 |  | 00 | 1.00 [.87, 1.15] | .977 |
| PSYDISC | – | – | – |  | -.59 | .55 [.36, .85] | .007 |  | – | – | – |
| Social distance | – | – | – |  | – | – | – |  | -.21 | .81 [.61, 1.09] | .164 |
| Spatial distance | – | – | – |  | – | – | – |  | -.13 | .54 [.63, 1.24] | .469 |
| Hypothetical distance | – | – | – |  | – | – | – |  | -.62 | -.62 [.35, .82] | .004 |
| Temporal distance | – | – | – |  | – | – | – |  | .15 | 1.16 [.86, 1.55] | .328 |
| Nagelkerke pseudo *R^2^* | .14 | | | | .17 | | |  | .21 | | |
| {\displaystyle \chi }χ*^2^ (df)* | 34.12 *(9), p* < .001 | | | | 41.74 *(10), p* < .001 | | |  | 50.08 *(13), p* < .001 | | |

*Note.* *N* = 436.

^a^ Gender: 0 = male, 1 = female

**Table S18**

*Predictive power of PSYDISC for COVID-19 vaccination hesitancy, Study 5*^[[4]](#footnote-4)^*.*

|  | Step 1 | | | Step 2a | |  | Step 2b | |
| --- | --- | --- | --- | --- | --- | --- | --- | --- |
|  | *b* [95% CI] | *p* |  | *b* [*95% CI*] | *p* |  | *b* [*95% CI*] | *p* |
| Gender ^a^ | .48 [.02, .23] | .051 |  | .53 [.05, 1.01] | .032 |  | .53 [.05, 1.02] | .031 |
| Age | -.04 [-.19, .02] | <.001 |  | -.04 [-.05, .11] | .000 |  | -.04 [-.06, -.03] | <.001 |
| Education | -.003 [-.14, .08] | .935 |  | .03 [-.16, .21] | .432 |  | .04 [-.04, .12] | .339 |
| Religiosity | .03 [-.18, .12] | .741 |  | .03 [.05, 1.02] | .791 |  | .03 [-.16, .21] | .764 |
| Spirituality | .10 [.02, .34] | .277 |  | .09 [-.10, .27] | .350 |  | .07 [-.11, .26] | .444 |
| Conspiracy beliefs | .19 [.02, .26] | <.001 |  | .18 [.07, .29] | .002 |  | .19 [.07, .30] | .002 |
| Political ideology | .15 [.01, .30] | .042 |  | .13[.05, 1.02] | .084 |  | .15 [-.001, .30] | .052 |
| Science knowledge | -.18 [-.16, .07] | .016 |  | -.15 [-.30, .00] | .057 |  | -.14 [-.29, .02] | .082 |
| Science understanding | .04 [-.22, -.004] | .485 |  | .07 [-.03, .17] | .184 |  | .07 [-.03, .18] | .173 |
| PSYDISC | – | – |  | .54 [.23, .85] | .001 |  | – | – |
| Social distance | – | – |  | – | – |  | .28 [.08, .48] | .005 |
| Spatial distance | – | – |  | – | – |  | .03 [-.22, .27] | .814 |
| Hypothetical distance | – | – |  | – | – |  | .20 [-.15, .55] | .253 |
| Temporal distance | – | – |  | – | – |  | .04 [-.17, .24] | .728 |
| Nagelkerke pseudo *R^2^* | .18 | | | .21 | |  | .21 | |
| -2 log likelihood=-/-=12- | 830.47 (*p* < .001) | | | 818.42 (*p* < .001) | |  | 815.54 (*p* < .001) | |

*Note. N* = 386, due to the question being asked to vaccinated individuals only.

^a^ Gender: 0 = male, 1 = female

1. Science knowledge scale contains two items measuring evolution knowledge. When these items are excluded, science knowledge is not a significant predictor in Study 2, although it remains significant in Study 1. [↑](#footnote-ref-1)
2. Science knowledge scale contains two items measuring evolution knowledge. When these items are excluded, science knowledge is not a significant predictor in Study 2, although it remains significant in Study 1. [↑](#footnote-ref-2)
3. Accounting for study in which participants previously participated by including it as a random intercept in a mixed logistic model did not alter the results. Variance attributed to the study was nearly zero. [↑](#footnote-ref-3)
4. Accounting for study in which participants previously participated by including it as a predictor did not alter the results, so we show the simpler model. [↑](#footnote-ref-4)
